# How does binding of agonist ligands control intrinsic molecular dynamics in human NMDA receptors?

**DOI:** 10.1371/journal.pone.0201234

**Published:** 2018-08-03

**Authors:** Zoltan Palmai, Kimberley Houenoussi, Sylvia Cohen-Kaminsky, Luba Tchertanov

**Affiliations:** 1 Centre de Mathématiques et de Leurs Applications (CMLA), ENS Paris-Saclay, CNRS-UMR 8536, Cachan, France; 2 Laboratoire d’Excellence en Recherche sur le Médicament et l’Innovation Thérapeutique (LabEx LERMIT), DHU TORINO (Thorax Innovation), INSERM UMR-S 999 - Université Paris- Saclay – IPSIT, Hypertension Artérielle Pulmonaire: Physiopathologie et Innovation Thérapeutique, Hôpital Marie Lannelongue, Le Plessis-Robinson, France; University of Parma, ITALY

## Abstract

NMDA-type glutamate receptors (NMDAR) are ligand-gated ion channels that contribute to excitatory neurotransmission in the central nervous system. NMDAR dysfunction has been found to be involved in various neurological disorders. Recent crystallographic and EM studies have shown the static structure of different states of the non-human NMDARs. Here we describe a model of a human NMDA receptor (hNMDAR) and its molecular dynamics (MD) before and after the binding of agonist ligands, glutamate and glycine. It is shown that the binding of ligands promotes a global reduction in molecular flexibility that produces a more tightly packed conformation than the unbound hNMDAR, and a higher cooperative regularity of moving. The ligand-induced synchronization of motion, identified on all structural levels of the modular hNMDA receptor is apparently a fundamental factor in channel gating. Although the time scale of the MD simulations (300 ns) was not sufficient to observe the complete gating event, the obtained data has shown the ligand-induced stabilization of hNMDAR that conforms the “going to be open state”. We propose a mechanistic dynamic model of the ligand-dependent gating mechanism in the hNMDA receptor. At the binding of the ligands, the differently twisted conformations of the highly flexible receptor are stabilized in unique conformation with a linear molecular axis, which is a condition that is optimal for pore development. By searching the receptor surface, we have identified three new pockets, which are different from the pockets described in the literature as the potential and known positive allosteric modulator binding sites. A successful docking of two NMDAR modulators to their binding sites validates the model of a human NMDA receptor as a biological relevant target.

## Introduction

N-methyl-D-aspartate receptors (NMDARs) belong to a family of L-glutamate ionotropic receptors (GluRs) that form the heterotetrameric ligand-gated channels located at cell-cell contact sites particularly for excitatory neuronal synaptic communication in the central nervous system (CNS) (for review, see [1]). The NMDAR consists of two obligatory GluN1 chains, and two GluN2 chains (from GluN2A, GluN2B, GluN2C and GluN2D) modulating channel properties, or a GluN2/GluN3 subunit combination. It is activated by glutamate (E) and glycine (G) or D-serine, in membrane depolarization conditions. Glutamatergic communication through N-methyl-D-aspartate receptors (NMDARs) in the CNS plays a major role in neuron fate and in learning and memory, but such signaling may also occur outside the CNS [2], including the vascular system [3]. NMDAR-mediated glutamatergic communication is dysregulated in neurodegenerative disorders, such as Alzheimer’s, Parkinson’s and Huntington’s diseases, and in stroke, in which NMDAR subunits undergo transcriptional and/or posttranslational modifications [1]. NMDAR has also been suggested to play a pathological role in chronic peripheral disorders, such as type 2 diabetes mellitus [4] and cancer [5]. In animal models of cancer, NMDAR-specific antagonists inhibit cancer cell proliferation, greatly increasing animal survival by preventing tumor growth [5]. Recently it has been shown that the dysregulation of glutamatergic communication via NMDARs between pulmonary vascular cells is involved in the lung vascular remodeling leading to Pulmonary Arterial Hypertension (PAH) and could be targeted pharmacologically to reverse established pulmonary hypertension in animals [6]. Thus, the targeting of peripheral NMDARs with specific antagonists may be beneficial in these conditions. In addition to the huge ongoing research performed to develop novel NMDAR antagonists to target diseases in the CNS [7] peripheral NMDAR antagonists that do not cross the blood brain barrier are under development (WO/2017/017116, WO/2017/093354, WO/2017/216159). Therefore a dynamic molecular model of channel opening of the NMDAR and the characterization of channel residues involved in the docking of open channel blockers is of the utmost interest to develop novel NMDAR antagonists, particularly, but not only, those that do not cross the blood brain barrier.

**Fig 1:**
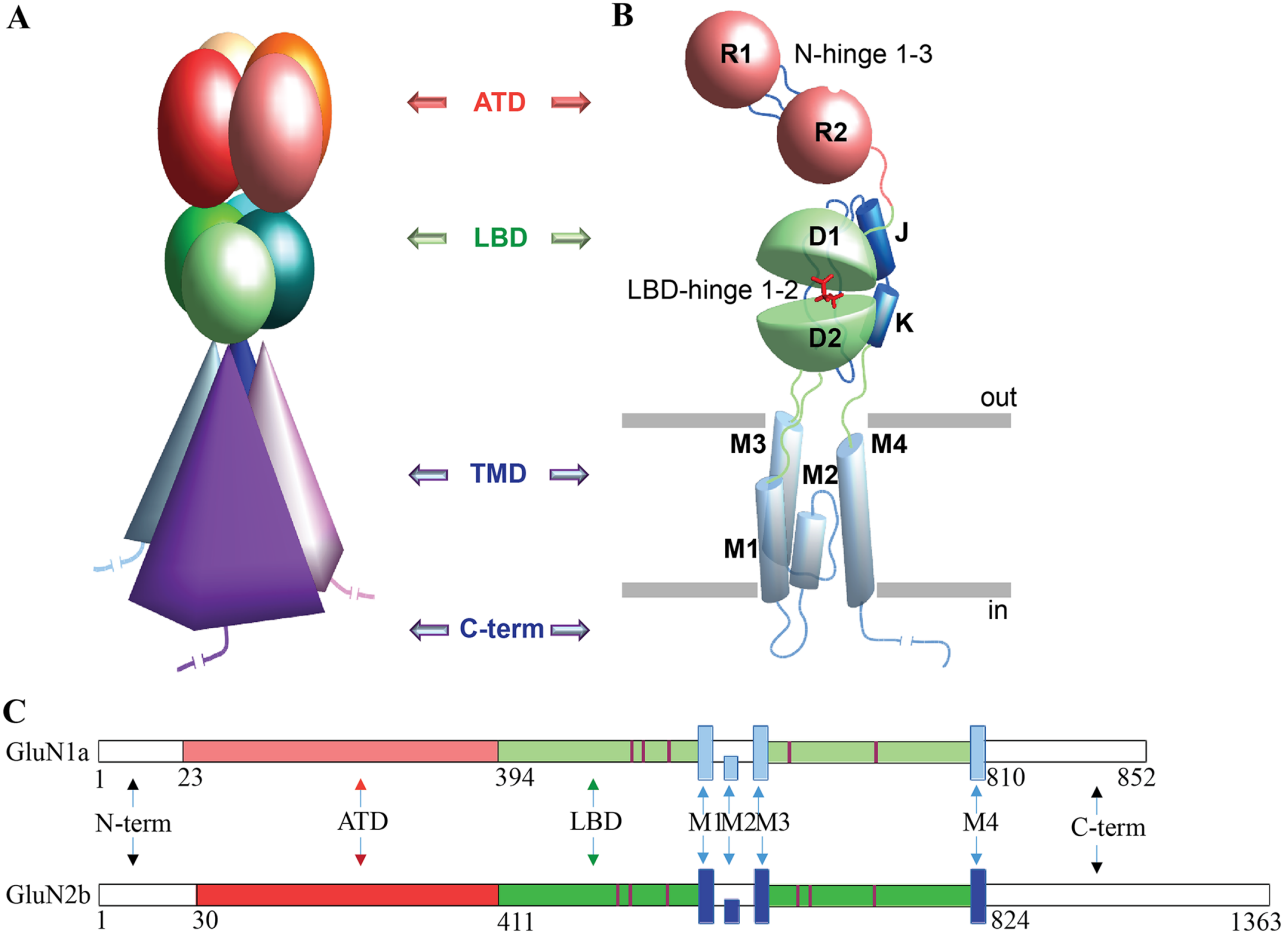
Architectural organisation and topology of NMDA receptors. (**A**) A tetramer modular complex is formed with two GluN1 and two GluN2 chains schematized in (**C**). (**A**-**C**) Each chain is composed of the amino-terminal domain (ATD), the ligand-binding domain (LBD), the transmembrane domain (TMD) and the intracellular C-terminal domain (CTD). The ATD consists of two lobes, R1 and R2, connected by N-hinge 1–3, which is linked by a loop with the two lobes (D1 and D2) of LBD that is in turn coupled with the TMD formed by four helices, M1-M4. Structural domains in **A**-**C** are distinguished by colors that vary within the same spectral region for each domain denoting different chains. Ligand (G) in the LBD binding cleft is shown using red sticks.

Despite having different roles in the activation mechanisms and distinct sequence composition and length, the NMDAR chains share structurally and functionally conserved structural domains consisting of the extracellular region composed of the amino-terminal domain (ATD) and the ligand-binding domain (LBD), the transmembrane domain (TMD) and the intracellular C-terminal domain (CTD) (Fig 1).

Each domain of this modular receptor performs its specific functional roles and contributes to a well-coordinated complex system, accomplishing the regulation of cation transport between extracellular and cytoplasmic matrices of a cell (a flux of Ca^2+^ and Na^+^ into the cell and K^+^ out of the cell throughout the TMD) [8]. The other role of NMDAR relates to performing the modifications (by CTD) of a variety of proteins with the kinase or phosphatase functions [9,10]. All these NMDAR functions are highly coupled. For instance, the increased concentration of Ca^2+^ turns off a switch for various signalling cell pathways [11]. The ATD influence the probability and promptness of activation/deactivation events [12] and the capacity of this domain to bound allosteric modulators [13], both suggest an emphatic contribution to the activation process of the ATD along with the LBD, promoting the channel opening.

The crystallographic structures of NMDAR for *Rattus norvegicus* (4PE5) [14] and *Xenopus laevis* (4TLL) [15], have revealed important insights into receptor architecture and structure in the inhibited state with a closed channel. Later, the structure of a limited resolution (cryo-EM) of *ratus* NMDAR in the inhibited and activated states is reported [16,17]. The coarse-grained modelling of *ratus* NMDAR has provided interesting information on the receptor dynamics during activation [18]. Combination of targeted molecular dynamics with application of the pore-lining helix repacking approach has generated a model of the open state of *Xenopus laevis* NMDAR [19]. However, despite the knowledge accumulated regarding NMDARs, the structure of human NMDARs has not yet been characterized. Moreover, the structural movements leading to the ion channel gating in NMDARs have not been fully described, and the receptor activation mechanisms are still unclear.

The available structures have enabled us to generate a structural model of the human NMDA receptor (hNMDAR) and use the all-atom molecular dynamics (MD) simulations to study the effects induced by simultaneous binding of two agonists to the receptor structure and the dynamics. Analysis of the simulation data has shown that the binding of two ligands promotes important changes in the conformational dynamics of the receptor, as shown by (i) a global reduction in flexibility that produces a more tightly packed and stable conformation with respect to the unbounded state, and (ii) an alternation of motions in the structural domains leaded to a highly cooperative regularity of movement. Such ligand-induced alternation of molecular motion was identified on all structural levels of the modular receptor, within a lobe, chain or domain, and between multiple lobes, chains or domains. While the motion of the bound receptor demonstrates a greater degree of regularity, it is postulated that the binding of two agonist ligands − glycine and glutamate − extensively synchronizes the dynamics of hNMDAR required for channel gating. The search of the surface pockets has revealed their large variability in the unbounded state that occurs due to the high conformational flexibility. The number of pockets is restricted by the binding of the ligands to hNMAR. By analyzing the locations of the pockets three novel putative sites have been distinguished from others reported in the literature as potential and known positive allosteric modulator (PAM) binding sites.

## Results and discussion

### 3D models of hNMDAR and their general behavior in MD simulations

**Fig 2:**
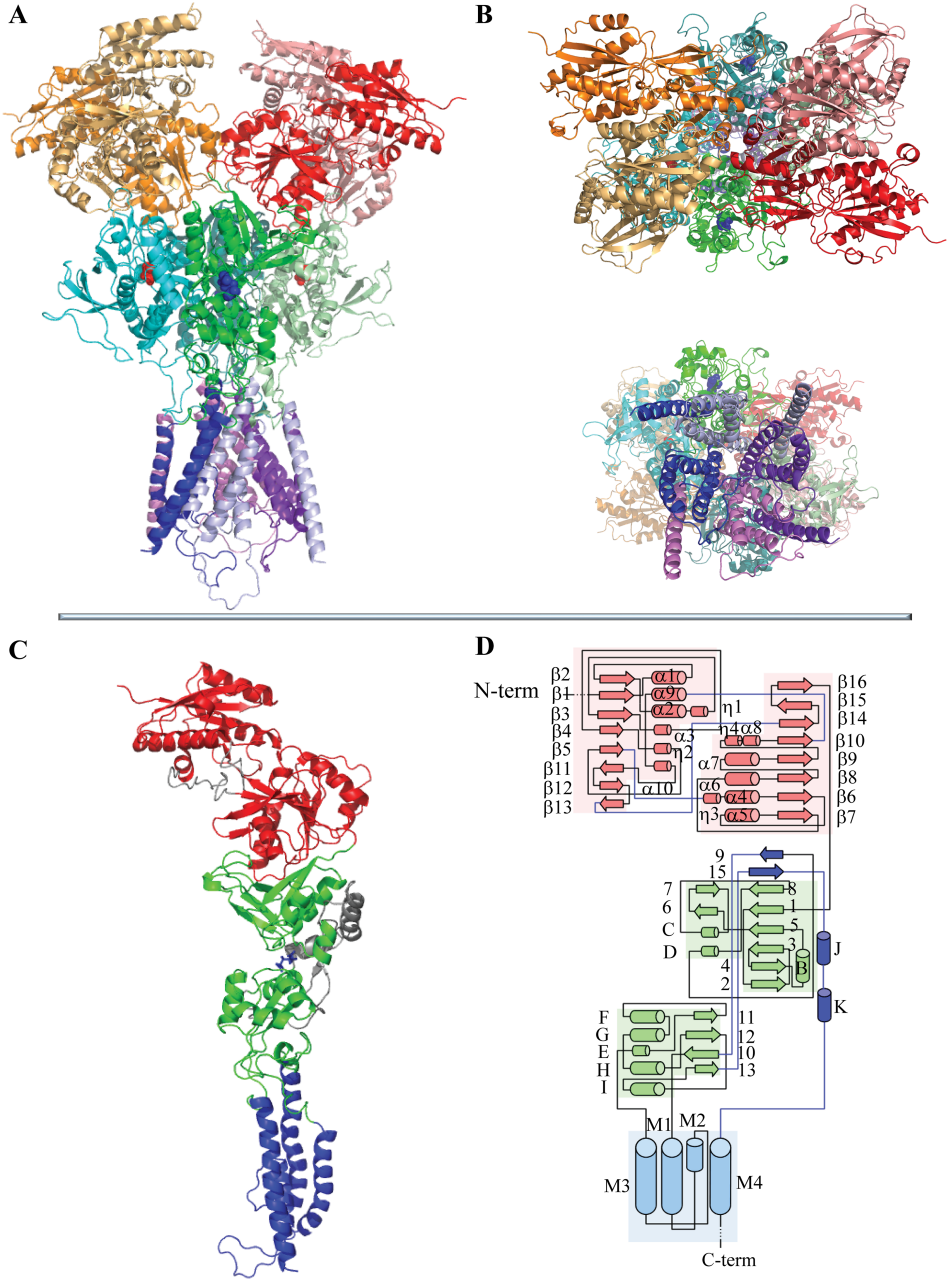
Structural model of hNMDAR. (**A-B**) Tetramer structure of hNMDAR, shown schematically, is viewed in three orthogonal projections; parallel to membrane, along the longest molecular axis (**A**) and in vertical orientation (**B**) with ATD (top) and TMD (bottom) in the foreground. (**C**) Structural fold of hNMDAR chains is shown for GluN1. Ligands E and G are shown as spheres (**A-B**) and as sticks (**C**). (**D**) Notification of the secondary structure elements is adapted from [14]. Structural domains in **A**-**D** are distinguished by colors with different shades for each chain within a given domain.

The available crystallographic data in the Protein Data Bank (PDB) [20] reporting the strongly modified (by deletion/insertion/replacement mutations and disulfide bridging) structure of NMDAR for *Rattus norvegicus* (pdb id: 4PE5) and for *Xenopus laevis* (pdb id: 4TLL, 4TLM) were used as templates for the construction of hNMDAR homology model. The template sequences are composed of GluN1/GluN2B chains. Considering the high similarity of GluN1/GluN2B sequences from human with those from rats and frogs (99.2/98.6 and 91.7/84.7% respectively), the GluN1 and GluN2B chains were chosen as the optimal composition for deriving the appropriate homology model of hNMDAR (Fig 2A and 2B). Since the GluN2B is widely distributed in the adult brain and has been reported in a range of disorders [21], this model will potentially be a key target of positive allosteric modulators [22]. Recently, it has been reported that GluN1 and GluN2B were found to be membrane components of all invasive adenocarcinoma and neuroendocrine pancreatic tumors [23].

The crystallographic structures 4PE5, 4TLL and 4TLM characterize the channel-closed state of NMDAR with a very similar LBD-open conformation. The root mean square deviations (RMSDs) calculated on the Cα-atoms of the G- and E-bound LBD for each pair of templates range between 0.78–1.16 Å. The RMSDs calculated on the Cα-atoms of M3 pore forming the trans-membrane helices are also showed small variations (0.85–1.01 Å). To build high-quality model of hNMDAR from sequence homology by a multi-template technique, we used these three structures to complete the structural information missing in each template.

**Fig 3:**
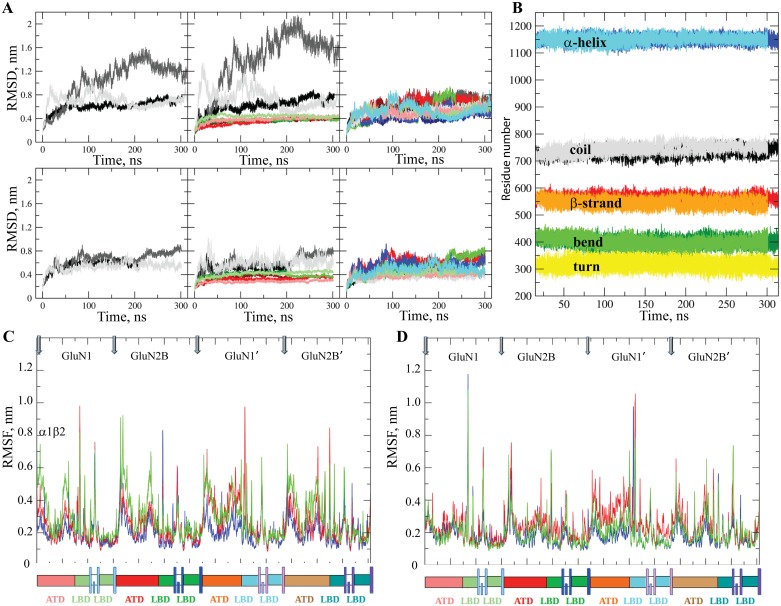
Molecular dynamics simulations of hNMDAR. (**A**) RMSDs from the initial model coordinates (t = 0 ns) are computed for all Cα atoms (left column), for the Cα atoms of each domain (middle column) and for the Cα atoms of each chain (right column) of hNMDAR (top) and hNMDAR•G•E (bottom). Trajectories **1–3**, structural domains and chains are shown with different colors. RMSDs calculated on all Cα atoms (left column) are shown in black, grey and light grey; on per domain Cα atoms (middle column) are shown in black, grey and light grey (ATD), in red, salmon and dark red (LBD) and green, dark green and light green (TMD); on per chain Cα atoms (right column) of GluN1, GluN2B, GluN1’ GluN2B’ are showed as shades of black, red, green and blue respectively. (**B**) Evolution of the secondary structure elements in hNMDAR (light colors) and hNMDAR•G•E (dark colors) was analyzed using the merged data (**1–3)**. The positions of the curves reflects the amount of secondary structure elements in the models, from small (turn) to big (α-helix). (**C-D**) The RMSFs values from the *average structure* coordinates were computed for all Cα atoms of the three independent trajectories, shown with different colors, and skipping the equilibration period of 15, 50, 85 ns for hNMDAR (**C**) and 15, 20, 25 ns for hNMDAR•G•E (**D**).

Since the structure resolution of NMDAR complexed with G and E (4PE5) is poor (3.96 Å), the 3D hNMDAR model was completed with agonist ligands, G and E, docked at their binding sites, which provides the generic hNMDAR•G•E complex. To create conditions close to the native environment of the receptor, each model, hNMDAR and hNMDAR•G•E, was embedded in phospholipid (POPC) bilayers to mimic the cell membrane and completed by water molecules and counter-ions. The produced systems were then explored using MD simulations over 300 ns with three independent runs to probe the conformational variability of proteins using random starting velocities. The simulations were initiated by a static homology model of a near full-length protein (missing the C-terminal), and we assessed and quantified conformational deviations from the model by superimposing each MD conformation on the homology model. The root mean square deviations (RMSDs) of hNMDAR and hNMDAR•G•E, which are computed using the positions of Cα atoms relative to the initial structure (t = 0) for each MD simulation run, range from 0.4 to 0.8 nm, with only one trajectory (hNMDAR) showing the RMSDs up to 1.6 nm (Fig 3A). Residues of ATD make the greatest contribution to the ample overall RMSDs (middle column). The RMS deviations within each full-length chain are not significant (right column).

Assignment of the protein’s secondary structure using DSSP [24] indicated well-defined and long-lived secondary structure elements–α-helices, β-strands, turns, bends and coils − that were highly conserved (i) over the simulation time and (ii) upon the ligand bindings (Fig 3B). Consequently, the structural content of both simulated systems is comparable and generally similar to structural templates, as is accurately explained in [14,15].

Profiles of the root mean square fluctuation (RMSF), describing the fluctuations of the Cα atoms with respect to the *average structure* show a series of sharp narrow peaks with large fluctuations for short molecular fragments, which are mainly the loops that connect the lobes or the domains (Fig 3C and 3D). More extended regions of hNMDAR including the ATD and LBD lobes exhibit high overall fluctuations. The periodicity in the RMSF pattern, which is expected due to the structural similarity of the chains, was observed in the both states of hNMDAR. Highly similar peak distributions of RMSF were observed between sequentially identical chains—and to a lesser extent—between heterogeneous chains.

The RMSD and RMSF profiles suggested that it may be possible to merge the MD simulation data collected during the three independent trajectories and analyze them together, skipping the equilibration periods.

### Conformational sampling

To investigate the conformational diversity of hNMDAR and hNMDAR•G•E during the MD simulation and make comparisons, the generated data has been analyzed using a multidimensional scaling analysis − clustering and principal component analysis (PCA). Clustering is the most suitable computational technique for dividing MD conformations into structurally homogeneous groups and quickly understanding the resulting sets [25]. In this approach, all MD conformations are sub-divided into several groups using a measure of similarity/dissimilarity. MD conformations that are placed in the same group are, according to some criterion, similar to each other and divergent from the conformations of other groups.

**Fig 4:**
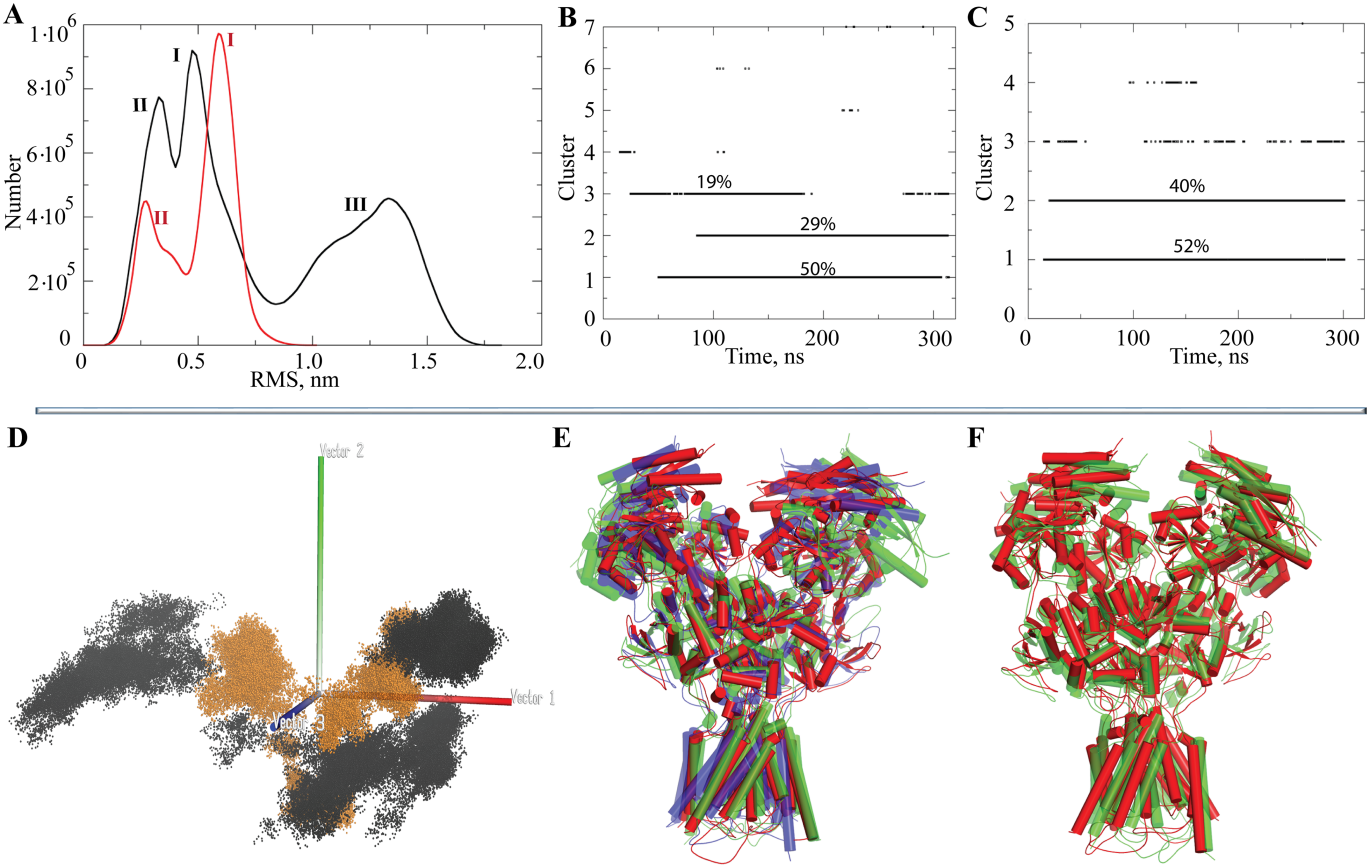
Analysis of MD conformations of hNMDAR and hNMDAR•G•E. (**A**) Distribution of the RMSD (Cα coordinates) of MD conformations of hNMDAR (black) and hNMDAR•G•E (red) measured from the *reference structure*. The clusters of conformations are defined with a cutoff of 4.5 Å in hNMDAR (**B**) and in hNMDAR•G•E (**C**). (**D**) Projection of hNMDAR (black) and hNMDAR•G•E (orange) MD conformations to the principal component (PC) subspace. (**E**) The superimposed *representative conformation*s of hNMDAR for cluster **1** (in red, cleft-closed ATD), **2** (in green, cleft-open ATD) and **3** (in blue, cleft-closed ATD, TMD twisted). (**F**) The superimposed representative conformations of hNMDAR•G•E for clusters **1** (in red, cleft-closed ATD) and **2** (in green, cleft-closed ATD, TMD twisted).

For each ensemble of conformations the cumulative distribution was estimated by calculating the Cα-RMSD with respect to the *reference structure*. The hNMDAR conformations are distributed in three distinct peaks; two of them (**I** and **II**), which show the proximal RMS values and means, are strongly overlapped (Fig 4A). The third peak (**III**), that is smoother and flatter, is mostly seen at large RMSD values. The hNMDAR•G•E conformations form a bimodal distribution with a sharp (leptokurtic) peak (**I**) and a smaller peak with a smoothed profile (platykurtic) (**II**), having smaller RMSD values with respect to the sharper peak (**I**).

The MD conformations of hNMDAR and hNMDAR•G•E were clustered to form structurally homogeneous subsets using an RMSD cutoff of 4.5 Å. It was found that MD conformations of the unbound and bound receptor were grouped into three and two main clusters (subsets), respectively (Fig 4B and 4C). In the unbound receptor subset **3** is composed of conformations, which were observed over the first half of the simulations, while the two other subsets were comprised of two distinct conformations that were detected everywhere over the 70–300 ns range of MD trajectories. The population of these individual clusters is of 50, 29 and 19% in **1**, **2** and **3** respectively. In the bound receptor, the two most populated clusters apparently represent two distinct conformations occurring with slightly different probabilities (52 and 40%) throughout the simulations.

Visual examination of the contents of the peaks and clusters showed that the flattened and wide Gaussian peak (**III**, with the largest RMSD values) in the distribution of conformations of unbound hNMDAR (Fig 4A) is composed of the ‘cleft-open ATD’ conformations (in green, Fig 4E), which are also systematically observed in cluster **2** (Fig 4B). The two overlapping peaks (**I** and **II**), with close RMSD values and means, represent two molecular conformations showing the similar ‘cleft-closed ATD’ conformations in which the channel axis of TMD is either parallel (**I**, in red) or not parallel in respect to the principal molecular axis (**II**, in blue) (Fig 4E). Similarly, the two-peak distribution of bound receptor contains two conformations that show similar ‘cleft-closed ATD’ conformations in which TMD is either rotated (in green) or not rotated (in red) around the channel axis (Fig 4F).

Previous studies have shown principal component analysis (PCA) to be an informative tool for the characterization of protein conformations obtained from MD simulations [26]. The principal components (PCs) were determined using PCA, and MD trajectories were projected onto the PC subspace. Projections of MD conformations on the subspace spanned by the first three eigenvectors indicated that the hNMDAR conformations were trapped in three separate regions that were localized in a large space, while the conformations of hNMDAR•G•E were grouped in two closed regions (Fig 4D).

Analysis of the simulation data using independent statistical techniques derived coherent results, which showed three distant conformations of the unbound receptor and two conformations in bound form. The RMSD distribution profiles and the clusters formed indicate that the modular structure of hNMDAR does not exhibit a limited number of discrete states but rather a continuum of conformations. Nevertheless, it can be supposed that some states may be energetically more favorable. The binding of two ligands to hNMDAR promotes a considerable reduction in the conformational space explored by the receptor during MD simulations, producing a less flexible and a more tightly packed conformation.

### Intrinsic internal dynamics

Since the NMDA receptor is a multi-domain and multi-chain protein, it is essential to describe the motions in local structures (*e*.*g*., local motions in a lobe), in domains (inter-lobe motions) and in the entire molecule (inter-domain motions). In order to explore the hNMDAR dynamic properties, the following questions are posed: (1) Which domain/fragment motions dominate the hNMDAR dynamics? (2) Are the intrinsic dynamic properties equivalent in hNMDAR and hNMDAR•G•E? (3) How does the binding of the ligands influence the motion of the structural domains and/or chains? Special interest was paid to the analysis of the interdependence of these motions.

**Fig 5:**
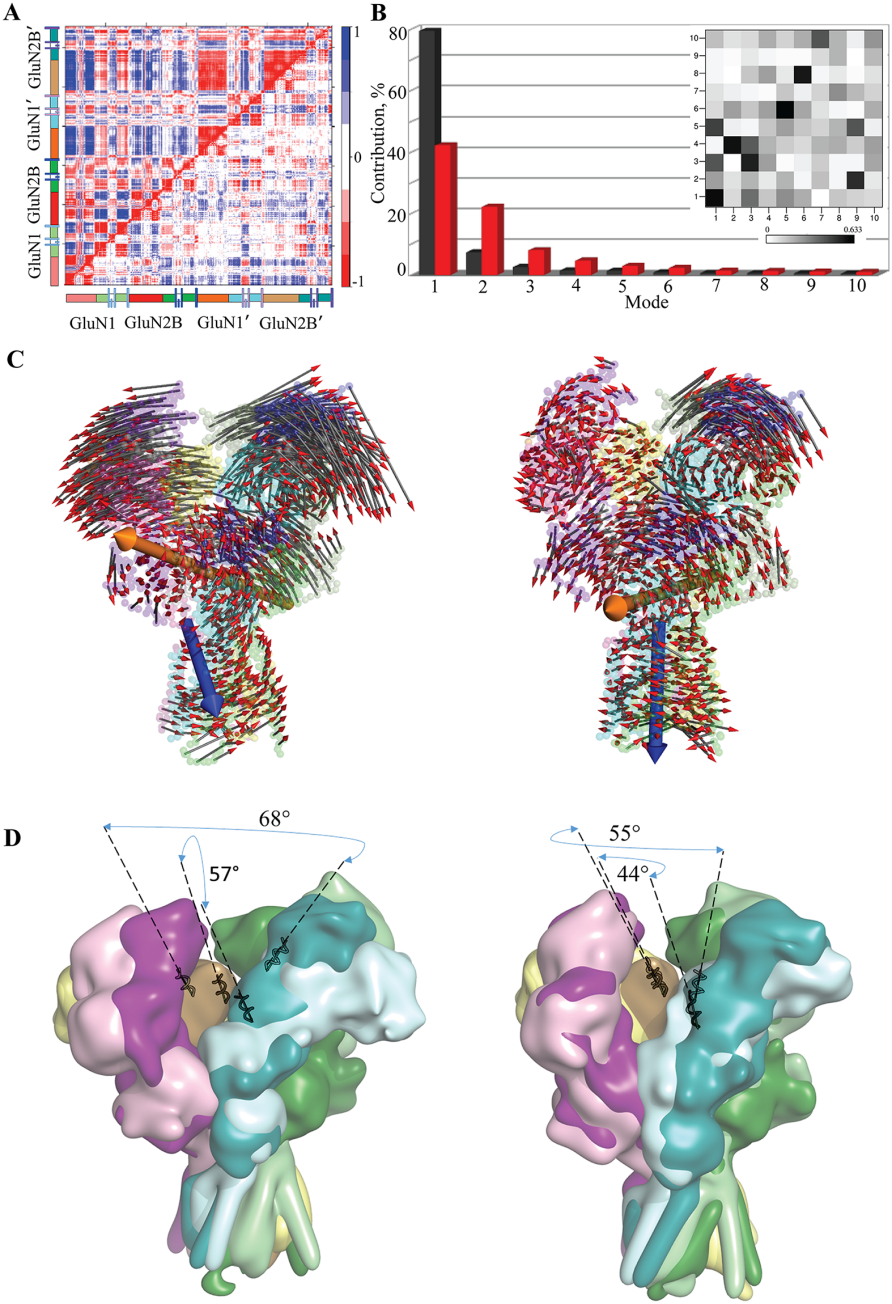
Slow motions in hNMDAR and hNMDAR•G•E. (**A**) Pearson cross-correlation maps from the merged trajectories (**1–3**) of Cα atom displacements of hNMDAR (upper left half-matrix) and hNMDAR•G•E (lower right half-matrix) after removing the overall rigid body motions of each receptor. The chains and structural domains are shown on the left and at the bottom of the matrix. Correlated and anti-correlated motions between atom pairs are color-coded from red (positive) to blue (negative). (**B-D**) Principle component analysis (PCA) performed on the Cα-atoms from the merged trajectories (**1–3**). (**B**) The bar plot gives the contribution of each mode to total RMSF of hNMDAR (in black) and hNMDAR•G•E (in red), in descending order. The grid shows the overlaps between eigenvectors from hNMDAR (X axis) and hNMDAR•G•E (Y axis) (insert). The overlapping similarity of the two eigenvectors is evaluated as their scalar product and represented by a rectangle, from white (0) to black (0.63). (**C**) Slow motions (mode 1) are illustrated by small arrows projected on hNMDAR (left) and hNMDAR•G•E (right). Two large arrows indicate rotational axes of structural blocks within a domain (rigid body motion) in LBD (orange) and TMD (blue). (**D**) Superimposition of two extreme conformations ‒ the ‘cleft-open’ ATD (light colors) and the ‘cleft-closed’ ATD (dark colors) in hNMDAR (left) and hNMDAR•G•E (right) are represented as molecular surfaces.

In order to obtain preliminary information on hNMDAR motions, a residue-based pairwise cross-correlation matrix was calculated for each form of the receptor. The cross-correlation matrix of Cα atom displacements was calculated after a superposition of each MD conformation on the *reference structure*. Three distinct patterns are distinguished from the map of hNMDAR (Fig 5A, upper left map): the first, a fractal-like pattern, shows strong correlations between size-restricted fragments (lobes, loops) within the same chain and between chains of GluN1-GluN2B heterodimer. The second pattern is mainly composed of large-size blocks that demonstrate strong positive correlations between extended structural fragments, composed of the ATD chains of GluN1′-GluN2B′ dimer or the blocks combining ATD and LBD lobes. The third pattern is a mixture of these two patterns showing strong anti-correlations between the two heterodimers. Consequently, the cross-correlation map of hNMDAR indicates highly coupled motions between (i) distinct structural domains within the same chain (inter-domain, intra-chain), (ii) similar structural domains of different chains (intra-domain, inter-chain), (iii) distinct structural domains of different chains (inter-domain, inter-chain) and (iv) molecular subdomains or fragments within a domain of the same chain (intra-domain, intra-chain). The correlation pattern of motion in hNMDAR•G•E is comparable with those in the unbound receptor, but the strength of the relationships between the domains is considerably reduced (Fig 5A, lower right map). The cross-correlation pattern obtained for each individual replica was in general similar to those for the concatenated trajectories (S1A Fig).

The overall architectural features of hNMDAR, which is composed of the four chains having a modular structure (Fig 1), are authentically reflected in these correlation patterns (Fig 5). The distinct patterns of the relationships within GluN1-GluN2B and within GluN1′-GluN2B′ may demonstrate non-identical motions in the two heterodimers.

Strongly correlated motions of lobes in the GluN1-GluN2B dimer of hNMDAR were observed by focusing on the correlations within each chain. Each lobe may be regarded as a dynamic unit exhibiting noticeable positive correlations within the domains of ATD or LBD, while between the domains their movements are anti-correlated. The helices from TMD also show highly correlated positive movements within the domain. In GluN1-GluN2B of ATD, the loops connecting the lobes demonstrated an alternation in sign of the motion correlation with respect to the lobes. Regarding the GluN1’-GluN2B’ heterodimer, it was observed that the lobes and the loops in ATD move together. Moreover, this movement involves the LBD lobes, producing a highly synchronized ample movement of two structural domains with respect to the other heterodimer in which the motions of ATD and LBD are disconnected.

Comparison of the two heterodimers revealed an anti-correlated motion of the ATD lobes of one dimer with respect to the other in both states, showing this effect strong for hNMDAR and moderate for hNMDAR•G•E. In contrast, the motion of the LBD lobes in each heterodimer demonstrated a common moderate positive correlation for both the unbound and bound forms. The helices from the TMD of two heterodimers showed highly correlated (positive) movement in each form. Such extensive cross-correlation patterns for both states of the receptor demonstrate a high degree of concerted motions between the neighboring fragments (proximal sites) and between the distant sites in regard to their relative position in sequence or in space.

### Slow motions

The slow motions of the receptor in unbound and bound states were characterized using principal component analysis (PCA). Ten PCA modes were sufficient to describe 95 and 87% of the total backbone fluctuations of hNMDAR and hNMDAR•G•E, respectively (Fig 5B). The cumulative contribution of the first three PCA modes was 89 and 72% for unbound and bound hNMDAR, respectively. Noticeably, mode 1 of hNMDAR bears a significantly larger contribution (79%) than that of hNMDAR•G•E (42%), while mode 2 of the unbound state contributes slightly with respect to the bound receptor. Computed scalar products between the first ten PCA modes from the two proteins indicated that the correspondence is not straightforward between the two ensembles (Fig 5B, insert). Namely, modes 1 and 3, which are intrinsically accessible for hNMDAR, are closely maintained in hNMDAR•G•E, sharing a relatively high degree of similarity (63%). This means that the direction in configurational space has a comparable amount of freedom in both simulations. Modes 2, 5 and 6 in hNMDAR are collinear with respect to modes 4, 6 and 8 in hNMDAR•G•E, which shows a noticeable reordering in direction and amplitude.

The most dominant first mode was used to illustrate qualitatively the ample movements of hNMDAR within each structural domain (Fig 5C, left). The ATD showed the greatest mobility by demonstrating a movement of two heterodimers, GluN1-GluN2B and GluN1’-GluN2B’, in opposite directions which promoted a local (within ATD) blooming or cleft opening. This scissor-like ample motion delivers a large range of hNMDAR conformations and shows a distinct relative displacement of the two heterodimers.

Surprisingly, the motion of two heterodimers of unbound hNMDAR were asymmetric; one of the two dimers demonstrated greater displacement with respect to the principle axis of the molecule. The motion asymmetry was previously described in coarse-grained simulations of *ratus* NMDAR, composed of chains GluN1 and GluN2A, which demonstrated distinct motions in ATD [18].

A residual composition at the ATD interfaces of each heterodimer was analyzed and it was found that they are principally composed of positively and negatively charged amino acids (Lys, Arg, His and Asp), which are localized in GluN1 and GluN2B respectively. The oppositely charged residues form short H-bonds and multiple salt-bridges between the chains (S2 Fig). These strong non-covalent interactions maintain the tight interface between the R1 lobes of the two heterogeneous chains in both the unbound and bound states of hNMDAR. However, the distances between the same chains from the two distinct heterodimers were more pronounced (> 4 Å).

Each heterodimer that has tightly connected chains (level R1 lobes in ATD) demonstrates a collective motion and moves as a pseudo-rigid body. Both lobes of ATD, R1 and R2 move in the same direction within a heterodimer and in the opposite direction with respect to the other heterodimer (Fig 5A). Two extreme conformational subsets of the unbound form, the ′cleft-close ATD′ and the ′cleft-open ATD′ states, were quantified using the angle between the α5 helices of GluN2B chains, which varied from 57 to 68°, respectively (Fig 5D, left). These two extreme conformations were previously reported in the low-resolution crystallographic structures of *frog* NMDAR harboring (59°) or not (84°) an engineered disulfide bridge between the heterodimers [15].

The ATD motions of hNMDAR•G•E were considerably reduced, with a variation in the angle between the α5 helices of GluN2B chains ranging from 44 to 55° (Fig 5D, right). Along with the diminishing amplitude, the direction of motion was also altered. Instead of a dominant flip-side motion of heterodimers in hNMDAR, the binding of the agonist ligands promoted a circular movement within each lobe of the ATD and/or within the two-chain subdomain, manifesting twisting components (Fig 5C). In the unbound receptor, circular or rotational motion-components were present inside the lobes of ATD, as shown by the 2^nd^ and 3^rd^ modes, but their contribution to the total receptor motion remained minor (S1B and S1C Fig). Similarly, a rectilinear motion in the ATD domain of the bound receptor shown for the 3^rd^ mode is negligible as a component.

The LBD global motion in both receptors, hNMDAR and hNMDAR•G•E, demonstrates a lower amplitude with respect to that of ATD. The motions of all LBD chains in both receptors are collective and may be described as a pendulum-like reversible movement along a common virtual rotational axis (Fig 5C). The orientation of this axis differs in the two receptor forms, and is non-orthogonal to the principal axis of the molecule in an unbound receptor, and it becomes perfectly orthogonal in hNMDAR•G•E.

In both states, the amplitude of the highly correlated movements in the TMD is significantly lower compared to that of ATD. Although the absolute values of the TMD motions are comparable in the two receptor states, their directions alternate strongly. The residues of the TMD chains rotate around a virtual direction that either coincides (in hNMDAR•G•E) or does not coincide (in hNMDAR) with the principal molecular axis (Fig 5C).

A significant reduction in the magnitude of ATD motion in hNMDAR•G•E, together with the concerted alternation of the direction of movement in all structural sub-domains of ATD, LBD and TMD, showed that the binding of two ligands strongly affects the molecular dynamics of the receptor. The effects induced by the binding of the ligands to LBD were observed as local events manifested by ordering the rotational movement of the LBD lobes around a virtual axis and as the long-distance control of motions in distant domains. These long-distance effects were shown to considerably diminish the amplitude of ATD motion and ordering of the rotational movements in ATD lobes and TMD. Both the local and long-distance effects illustrate the manifestation of allosteric regulation of hNMDAR, which is the phenomenon controlling the activity of all membrane receptors and nearly all proteins [27]. Apparently, hNMDAR belongs to the proteins that achieve allosteric control through an alternation of dynamics rather than structural re-folding.

### Structural and dynamical features of LBD

**Fig 6:**
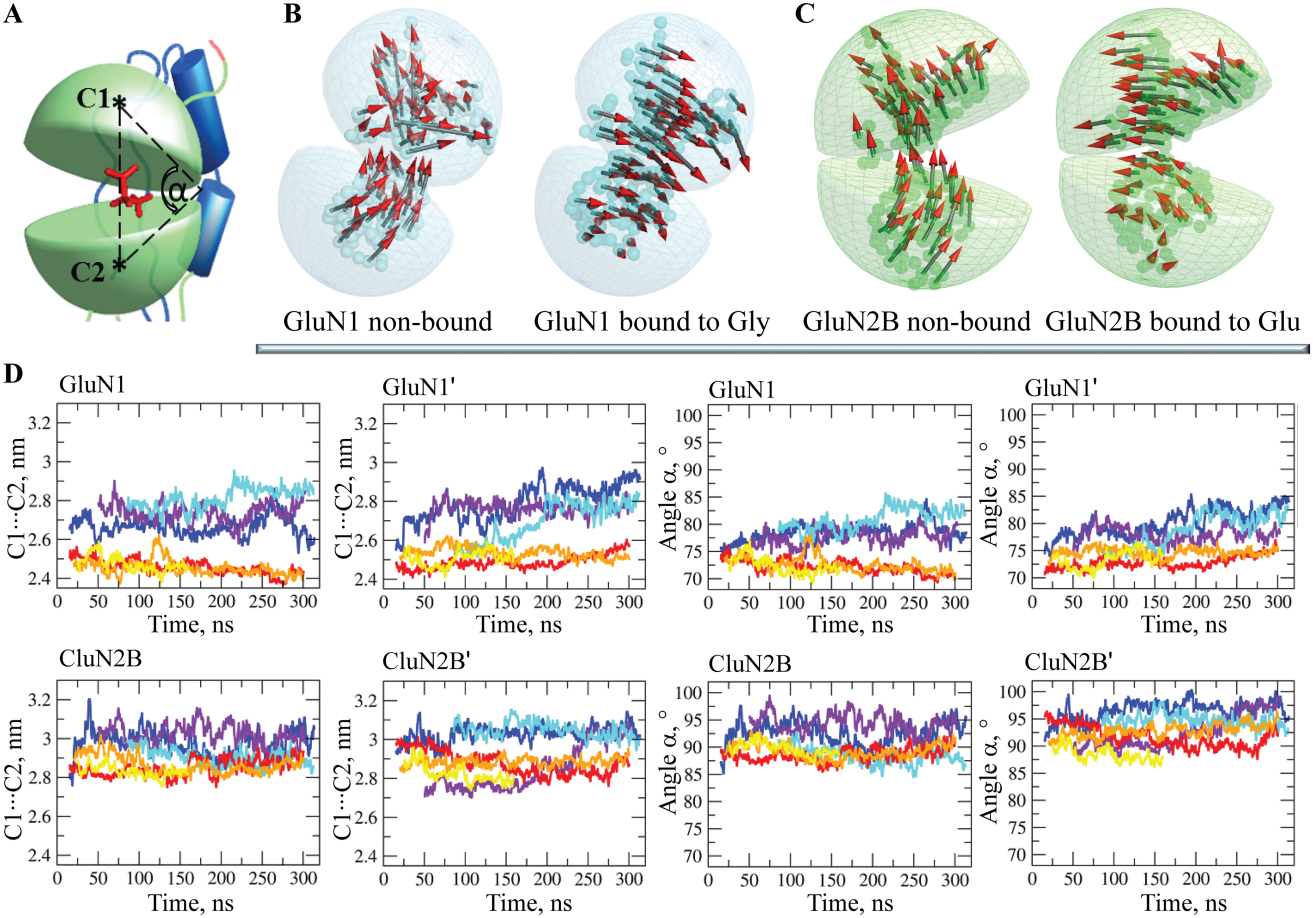
Structural and dynamical features in LBD of NMDA receptor. (**A**) Two lobes (green) of LBD with a ligand (red sticks) in the agonist-selective binding cleft. Centroids C1 and C2, defined on lobes D1 and D2, were used to calculate the LBD geometry. (**B, C**) The first PCA mode illustrates the slow motions by using small arrows projected on the LBD, which is depicted as a transparent spherical shape. Collective motions in the LBD are shown in two chains, GluN1 (**B**) and GluN2B (**C**) of hNMDAR (left) and hNMDAR•G•E (right). (**D**) The LBD geometry is characterized by the monitoring throughout the MD simulations of two metrics, the distance C1⋯C2 between two centroids (C1 and C2) defined on each lobe (D1 and D2), and the angle α formed by centroids C1, C2 and C3, where C3 is the centroid of the D1-D2 hinge. The metrics of non-bound and bound receptors are differentiated by color: blue, violet and cyan for hNMDAR and red, yellow and orange for hNMDAR•G•E.

The LBD of each chain consists of two hinged lobes, D1 and D2, which form a narrow agonist-selective binding cleft (Fig 6A). The LBD heterodimer interface is braced by specific contacts between the polar residues from the D1 and D2 lobes, which are Q696 from GluN1, N693 and N697 from GluN2B of the adjacent chains and Y535 of GluN1, with residues located at the D1-D2 hinge (S3B Fig). It has been proposed that these interactions regulate NMDA receptor deactivation kinetics by controlling the stability or geometry of the closed-cleft conformation [28]. Indeed, these residues are localized on a periphery of the lobes, close to the J-K hinges (Fig 2C and 2D). Q696 (GluN1) is in contact with the J-K hinge of GluN2B’; similarly, N693 and N697 (GluN2B’) are in contact with the J-K hinge of GluN1. Such cross-heterodimer interactions may control the hinge geometry. Y535 is positioned at the hinge between the four lobes and may play a crucial role in hinge bending.

Focusing on the principal motions in LBD (PCA), it was observed that the binding of G increased the degree of collectivity of the movements in both GluN1 chains, making the motions of D1 and D2 strongly concerted. Indeed, by comparing the PCA vectors (the 1^st^ mode) projected onto the D1 and D2 lobes of LBD, one can note a high degree of disorder in the vector directions in the unbound receptor, notably at the binding sites of G (GluN1 and GluN1’ chains) (Fig 6B). In the bound state, the PCA vectors of the D1 and D2 lobes are well ordered and parallel within each chain.

The vectors describing the directions of motion in the two lobes of chains GluN2B and GluN2B’ in the unbound receptor, are already well ordered and reflect a collective character of motion in both lobes. The E binding eventually improves the completeness of this concerted motion and promotes an alternation in its direction with respect to the hinge (Fig 6C). Consequently, the binding of glutamate and glycine to their binding sites increases the regularity of the atomic displacement in the same direction for GluN1 chains and alternates the direction of the collective motion within each GluN2B chain. Globally, such ordering and/or alternation of the lobe motions promoted an adjustment of the common virtual rotational axis in LBD that became perfectly orthogonal relative to the principle molecular axis.

The LBD geometry was characterized by two metrics, the distance C1⋯C2 between two centroids (C1 and C2) defined on each lobe (D1 and D2), and the angle α between centroids C1, C2 and C3 (Fig 6A). In all receptor chains, the distance C1⋯C2 and the angle α are diminished in hNMDAR•G•E with respect to the unbound receptor (Fig 6D), indicating a ‘closed-cleft’ LBD conformation that is stabilized by the binding of the ligands. The distances between the D1 and D2 interacting surfaces (the ligand binding residues of D1/D2 lobes), which are monitored throughout the MD simulations of unbound and bound forms of hMNDAR, indicate that the ligands have a bridging role by linking the two lobes (S3C and S3D Fig). The tight binding of the ligands to the clefts of hNMDAR promotes the strengthening of the D1-D2 interactions braced by unique contacts.

### Structural and dynamical features of the TMD

The channel gating as a response to the ligands binding is not instantaneous and requires a sufficiently long period of time (order of milliseconds) [29]. Obviously, this process is beyond the time scale used in our study. From the 300 ns MD simulations it was not expected that large conformational rearrangements of the channel pore promoted by the binding of ligands would be observed, however it was hoped that the comparative analysis of the conformational dynamics of the unbound and bound receptors would shed light on the effects induced by the binding of two ligands that lead to the channel opening.

The M3 segments of the TMD were reported as a crucial element in the activation pathway of NMDA receptors [30]. As it was suggested in [15], the residues T646 and A645 from the M3 helices form the bottleneck of the channel. To characterize the structure and dynamics of this segment, the related metrics from the PCA were obtained and compared between the free-ligand NMDAR and its complex bound to two ligands.

**Fig 7:**
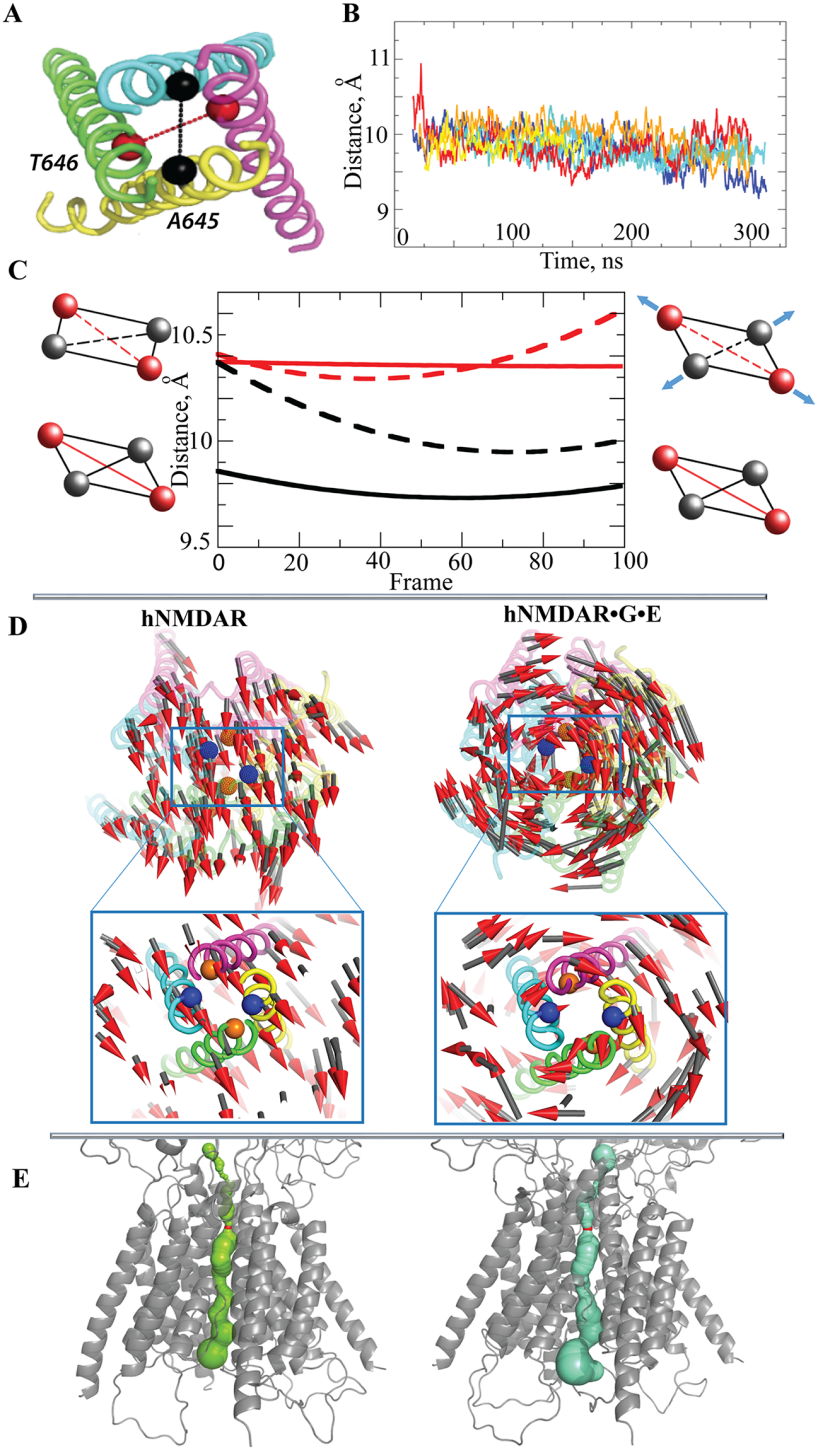
Structural and dynamical features of the TMD. (**A**) The M3 segment of the TMD formed by M3 helices of GluN1 and GluN1’ (green and pink), and GluN2B and GluN2B’ (cyan and yellow) chains. Residues shown as spheres are the T646 (red) and A645 (black), hypothesized by (Lee et al., 2014) as forming the bottleneck of the channel. (**B**) Distance between the Cα atoms from bottleneck residues T646 of two GluN1 chains in hNMDAR (blue, violet and cyan) and in hNMDAR•G•E (red, yellow and orange). (**C**) The bottleneck diameters, defined as distances between the Cα atoms of A645 from two GluN2B chains (black), of T646 from two GluN1 chains (red), are shown for hNMDAR (solid lines) and hNMDAR•G•E (dashed lines). Distances were measured along the 1^st^ PCA mode (for 100 frames in-between extreme projections, see Methods). Rectangle and parallelogram are used to demonstrate the shape formed by bottleneck residues from M3 helices. (**D**) The first PCA mode illustrates the slow motions in the TMD using small arrows projected onto the channel. (**E**) The channel pore in hNMDAR (left) and hNMDAR•G•E (right). The bottleneck is shown in red.

In order to measure the bottleneck diameter (the distance between the diagonally facing bottleneck residues, Fig 7A), the two extreme conformations along the first PCA eigenvector were projected onto the averaged structure with 100 interpolated conformations (frames) along the trajectory between these extreme conformations (Fig 7C). In the unbound receptor, the bottleneck diameters for the first PCA mode are strongly conserved during the 100 frames, where the distance between A645 residues is 9.8 Å (in black) and between T646 residues is 10.4 Å (in red), and this represents a flattened, ellipse-like (shown by a parallelogram) shape (Fig 7C, bottom, continuous diagonal parallelograms). Such conservation of diameters demonstrates that the shape of the bottleneck profile is maintained.

On the contrary, the extreme conformation of frame 0 in hNMDAR•G•E exhibits equal diameters (of 10.4 Å), while the other extreme conformation, frame 100, shows slightly different values, of 10.0 Å and 10.6 Å. Therefore, the two extreme conformations (frames 0 and 100) − along the first PCA mode − possess different pore shapes, an almost circular (schematically represented by a rectangle) and an ellipse-like shape (represented by a parallelogram) (Fig 7C, top, dashed diagonal rectangle and parallelogram). The difference in the diameters of the ellipse-like shape is equivalent in the two forms of the receptor, however the values of each metric (diameter) in the bound form is slightly increased with respect to the unbound. Based on these findings, one can conclude that the alteration of TMD rotation axis upon ligand binding flattens and rounds (depending on the direction of rotation) the bottleneck pore profile (Figs 5C and 7D). The similar analysis of the second and third PCA eigenvectors also shows the pore shape changes along each mode (S4 Fig).

Furthermore, the bottleneck diameters in the TMD were systematically calculated over the MD simulation using the distances between the Cα atoms of bottleneck residue A645 from two GluN2B chains, and of T646 from two GluN1 chains. Both distances were slightly diminished at the end of the MD simulations of hNMDAR, and conserved or increased in hNMDAR•G•E (Fig 7B). Analysis of the pore geometry using the angles formed by the M3 helices showed perfect conservation over the simulation time in both forms of receptor (S5 Fig). These results indicate that each helix in the M3 segment maintains its relative position with respect to the others. Nevertheless, the estimation of the pore bottleneck size by CAVER [31] in the most representative structures identified by clustering analysis, which took into consideration the side-chain of each bottleneck residue, resulted in slightly different values, of 1.7 and 2.3 Å for hNMDAR and hNMDAR•G•E, respectively (Fig 7E).

Focusing on the overall principal motions in TMD (PCA, 1^st^ mode), it was observed that in the unbound receptor, the M3 helices move together in the same direction demonstrating a rectilinear round trip (Fig 7D). The binding of ligands strongly reorganized the motion of the residues in the TMD, which moved in a circular orbit around the same virtual axis (U-turn motion). Apparently, the motion of all M3 helices in hNMDAR•G•E is highly synchronized and similar to the motion of a balance spring.

In summarizing the observations on the M3 segment measures and dynamics in the TMD over the simulation time, it is noted that in the unbound state, (i) the atomic displacements in the TMD are parallel and describe a rectilinear round trip; (ii) the bottleneck profile shape is maintained; (iii) the direction of global TMD motion does not coincide with the principal axis of the molecule. Upon the binding of two ligands (i) the atomic displacement constitutes a synchronic balance spring movement; (ii) the bottleneck profile shape alternates; (iii) the direction of the TMD rotational motion coincides with the principal axis of the receptor.

Despite the significant divergences in the TMD dynamics for the two receptor states, reconstitution of a pore across the whole TMD did not show a pronounced difference between the two forms of the receptor (Fig 7E), as was reported by [19]. Nevertheless, such an arrangement of the M3 segment geometry and dynamics may be interpreted as ‘closed’ in hNMDAR and a ‘going to be open’ pore profile in hNMDAR•G•E.

The relative conformation of residues R684 (GluN1) and E658 (GluN2B), localized in the interface region connecting the LBD and TMD, was reported as representative in distinguishing non-active and activated states of the receptor [16]. The geometry of the shape formed by these residues along the first PCA mode (for 100 frames in-between extreme projections, see Methods) was monitored. Both metrics, the distance between E658 residues of two GluN2B chains and the angle formed by the two E658 and a R684 of GluN1’, were constant along the first PCA eigenvector in unbound hNMDAR (S5 Fig). Whereas, the metrics differed significantly in the bound receptor. The conformation that was characterized using a longer distance and flattened angle (frame 0) describes the state defined as ‘going to be open’, while the conformation that has a shorter distance and sharpened linker angle (frame 100) refers to the ‘closed’ state. The data obtained from the simulations corresponds well with the values (measures) observed in the experimentally characterized structures of the non-active and activated states of NMDAR [16].

### Proposed mechanisms for channel gating

Despite the limited simulation time, the 300 ns data proved to be sufficient to characterize the conformational dynamics of the NMDA receptor and distinguishing between its unbound and bound forms. Alteration of the dynamic features was detected at all structural levels of the modular hNMDAR − within the lobes, domains and chains, and between these structural subunits.

**Fig 8:**
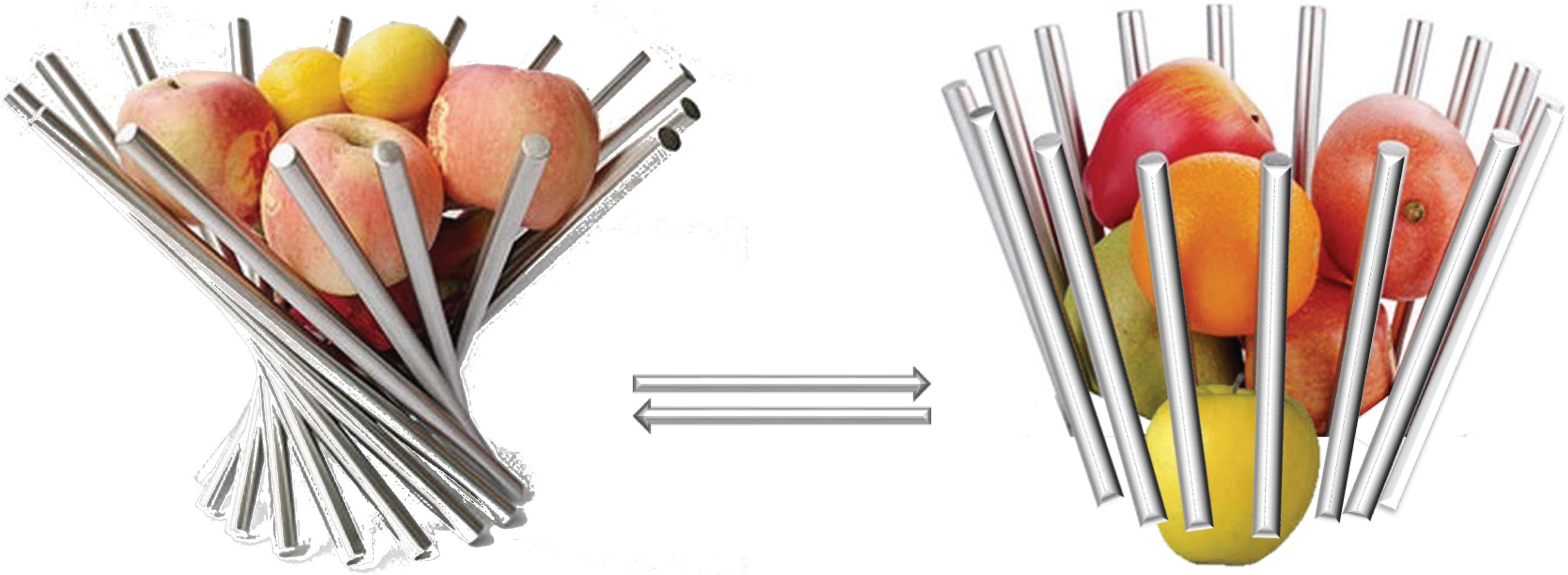
Metaphoric representation of channel gating in a hNMDAR receptor. The differently twisted conformations of the unbound state maintains the ‘closed’ channel (left), while the conformations of hNMDAR•G•E are well ordered and centered on the unique direction in the whole chain, which is coincident with the principle axis of molecule (right), stabilizing the channel ‘opening’. Transition between the two states is allosterically regulated by the binding of two agonist ligands, G and E, to the LBD.

In summarizing the obtained results, a mechanistic dynamic model of the gating mechanism in the receptor was hypothesized. In the free-ligand receptor, high flexibility of each chain and structural sub-domain delivered a large amount of NMDAR conformation. The different kinds of global movements of each domain − flip-side of heterodimers in ATD, pendulum-like in LBD and rectilinear round trip in TMD—produce a huge number of conformations in which the molecular axis is frequently non-linear (bent or twisted) and oriented differently in a space, as represented metaphorically in Fig 8, on the left. The TMD rotation occurs around an axis forming an arbitrary angle with the molecular axis.

In the receptor stimulated by the binding of two ligands, the rectilinear movement of the residues of each chain in all domains is considerably diminished, and the rotational component appears to be prevalent. The circular motion involves either the entire domain (as observed in the LBD and TMD) or subdomains (typically, in the ATD). The collective global motion is centered on a unique direction within a domain, demonstrating highly synchronized reversal movements − *pendulum-like* in the LBD and synchronic balance spring in the TMD. These motions are highly directional with respect to the principle molecular axis of hNMDAR − orthogonal in the LBD and parallel in the TMD. As a result, only the conformations with nearly linear molecular axes are stabilized, which is an optimal condition for pore development across the entire receptor. The channel pore may vary from the initial values to a larger size that is required for the flow of cations (Fig 8, on the right).

### Identification of pockets on the hNMDAR surface

Restricted target-related data is one of the elements that restrain the exploration of new molecules using structure-based approaches. The identification and characterization of small-molecule binding pockets are crucial factors in the search for hit compounds. Traditionally, the search for pockets is performed on crystallographic structures or on rigid models. MD simulations can be helpful in the discovery of new binding sites, through the exploration of thousands of protein conformations that describe the structural and dynamical behavior of macromolecules.

**Fig 9:**
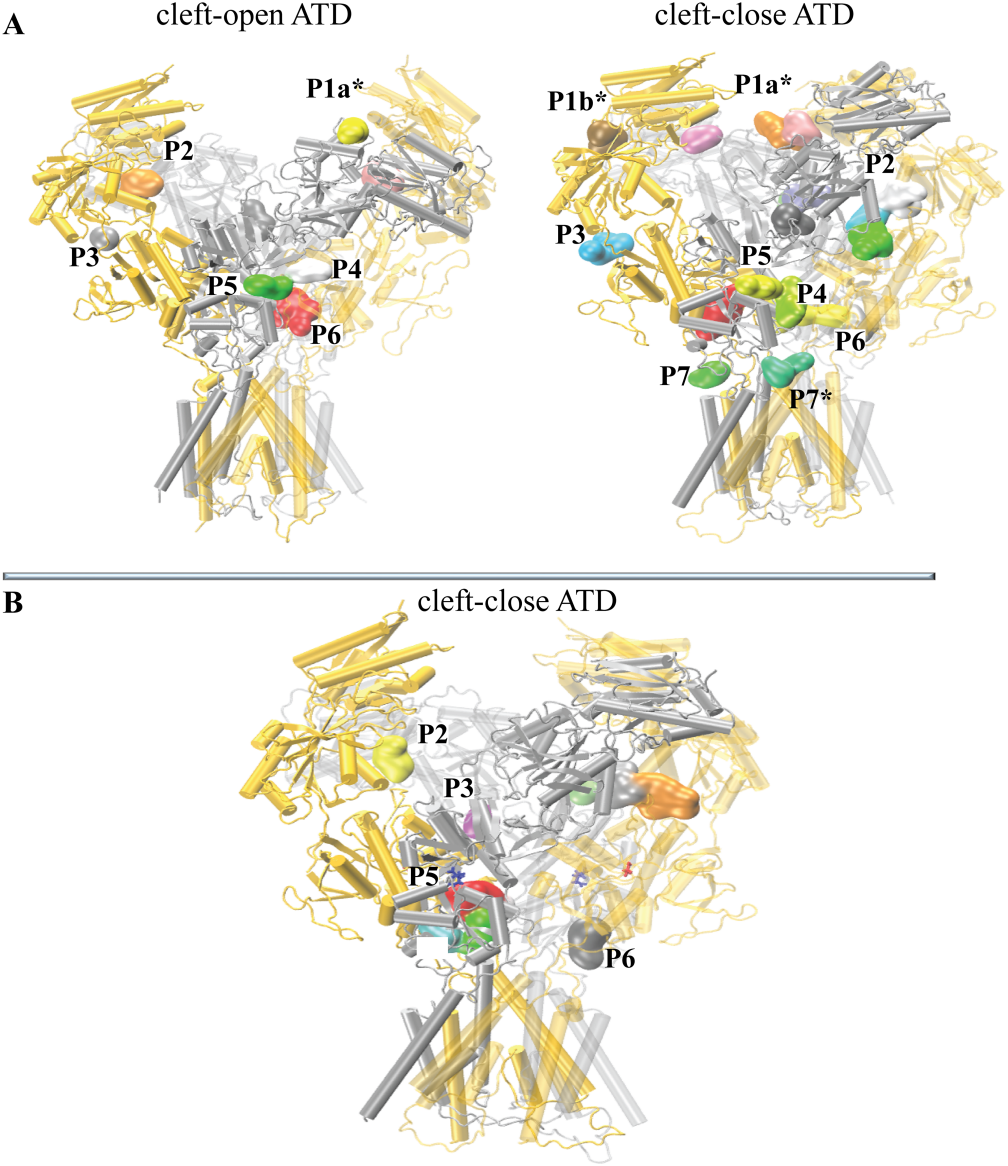
Pockets detected at the hNMDAR surface. (**A**) Two extreme conformations, qualified as the ′cleft-open′ ATD (left) and the ′cleft-close′ ATD (right) of the unbound hNMDAR. (**B**) The ′cleft-close′ ATD conformation is from the most populated cluster of hNMDAR•G•E. The protein is shown schematically with GluN1 and GluN2B chains in gold and silver respectively. The pockets (P) are differentiated by color and numerated. The pockets reported in the literature are referenced; an asterisk denotes the newly described pockets.

The protein surface in two extreme conformations of unbound hNMDAR, ‘cleft-open ATD’ and ‘cleft-closed ATD’, and the representative structure from the most populated cluster of conformations of hNMDAR•G•E was carefully investigated with *Fpocket* [32]. The number of identified pockets at the surface of the unbound receptor in the ‘cleft-close ATD’ conformation was greater (clustered in eight positions) than in the ‘cleft-open ATD’ conformation (localized in six positions) (Fig 9A). Some pockets (P1b* and P7*), that are well defined in the ‘cleft-close ATD’ conformation, were not observed in the ‘cleft-open ATD’ conformation.

The majority of the identified pockets were described in the literature as potential and known positive allosteric modulator (PAM) binding sites and were summarized in [22]. As such, the pockets P2 (Fig 9, in orange and in pink in the ‘cleft-open ATD’ conformation and in white in the ‘cleft-close ATD’ conformation) localized between the R2 lobes (in the upside-down ‘V’ hole) of GluN1 and GluN2B in each heterodimer were suggested as the binding site for spermine [33]. The pockets P3 (in grey in the ‘cleft-open ATD’ conformation and in blue, grey, green and purple in the ‘cleft-close ATD’ conformation), located at the interface between the ATD and LBD, were suggested as the binding site for PYD-106 [34]. The pockets P4 (in white in the ‘cleft-open ATD’ conformation and in lime green in the ‘cleft-close ATD’ conformation) positioned at the interface between GluN1 and GluN2B in the LBD were suggested as possible binding sites for UBP compounds [35].

The pockets P6 (in red in the ‘cleft-open ATD’ conformation and in red, yellow in the ‘cleft-close ATD’ conformation) located between the D2 lobes of GluN1 and GluN2B’ (red) and between D2 lobes of GluN1’ and GluN2B, were suggested as the binding site for GNE-6901; GNE-8324 [36, 22] and TCN-201 inhibitors [37]. The pockets P5 (in green in the ‘cleft-open ATD’ conformation and in yellow in the ‘cleft-close ATD’ conformation) located in cleft between the D1 and D2 lobes of the LBD in GluN2B represent the binding site of E.

The pockets at position P7*, which were only observed on the ‘cleft-close ATD’ conformation, are located at the interface between the LBD and TMD, above M1 of GluN2B (green) and above M4 of GluN2B (cyan). The first pocket (in green) was suggested as the binding site for CIQ [38], while the second (in cyan) is apparently a newly identified pocket.

Pocket P1a* was also identified and localized between the R1 lobes at the center of the ATD (in yellow in the ‘cleft-close ATD’ and in pink/orange in the ‘cleft-open ATD’ conformations respectively). Additionally, in the ‘cleft-close ATD’ conformation of the unbound receptor, pocket P2b* (in brown) was localized between the R1 and R2 lobes of GluN1. These pockets − P1a*, P1b* and P7* (in cyan) − are distinct from the other described pockets, and, to the best of our knowledge, represent newly discovered pockets.

The surface pockets on the most representative conformation of the bound hNMDAR, having a ‘cleft-close ATD’ with straight TMD conformation are localized at four positions (Fig 9B) reported earlier in literature.

Comparing the number and variability, *i*.*e*. the ‘richness’ of the available binding pockets in the unbound and bound forms of the receptor, one can note an important reduction in their number when binding of the two ligands occurs. It suggests that the binding of ligands to the LBD may have additional allosteric effects beyond channel gating, leading to a reduction in the number of pockets throughout the overall receptor structure and therefore introducing indirect competitiveness between ligands and potential modulators.

**Fig 10:**
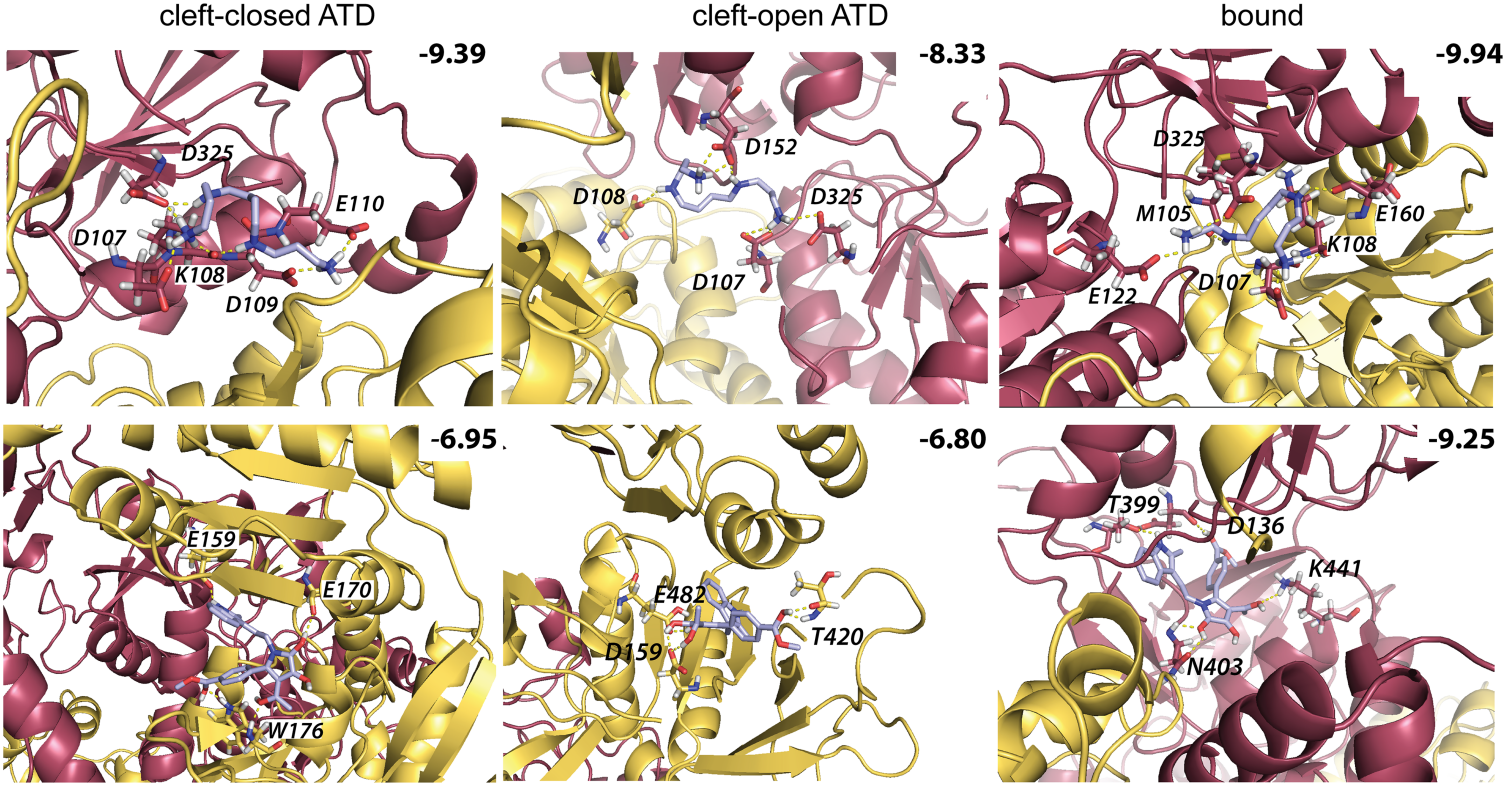
The modulators binding to hNMDAR. Docking poses of spermine in the binding pocket P2 (upper panel) and PYD-106 in the binding pocket P3 (bottom panel) of two extreme conformations of unbound hNMDAR (’cleft-open ATD’ and ‘cleft-close ATD’) and one of bound receptor. The protein is viewed as cartoon with GluN2B in red and GluN1 in yellow. Each modulator and protein residues contributing to the binding are shown in sticks. H-bonds are represented by dashed lines. The score values (kcal/mol) are indicated at the top of each docking pose.

To validate the generated model of hNMDAR, two different modulators − spermine, a NMDAR agonist, and PYD-106, a NMDAR positive allosteric modulator, bound in the pockets P2 [33] and P3 [34] respectively, − were docked using AutoDock into the two extreme representative conformations of unbound hNMDAR (’cleft-open ATD’ and ‘cleft-closed ATD’) and the one major representative conformation of bound receptor. The docking trials produced highly populated clusters demonstrating the unique pose of each molecule in its binding site. Spermine is localized between the R2 lobes of ATD and is mainly linked to GluN2B by strong and multiple H-bonds (Fig 10, top panel) demonstrating its GluN2B-selective character. The H-bonds of spermine with residues D107 and D325 are conserved in all conformations of hNMDAR. The docking scores indicated that NMDAR•G•E is better target of spermine than hNMDAR. PYD-106 bound to P3, located at the interface between the ATD and LBD, by at least 3 H-bonds (Fig 10, bottom panel). According to the docking scores, a higher affinity of PYD-106 is observed towards hNMDAR•G•E relative to the unbound form, in agreement with the experimental measurements [34].

## Conclusions and perspectives

NMDA receptors − the large tetrameric ligand-gated ion channels **−** have a unique modular architecture. Near-full-length structural models of human NMDAR have been built in the free-ligand (unbound) state and the bound states containing its natural ligands, glycine and glutamate. The dynamics of these models have been investigated using all-atom MD simulations. It is emphasized that the time scale of our simulations (300 ns) is not sufficient to observe the complete gating event; however, the differences between the two dynamic states of the receptor at all structural and architectural levels have been demonstrated. Comparison of the dynamic properties and geometry between these two forms of hNMDAR reveal that the bound hNMDAR assumes a more tightly packed and stable conformation than the unbound hNMDAR. It was demonstrated that the alternation in the motion induced by the binding of ligands was conducive to highly synchronized intra- and inter-domains movement, which is required for channel gating. Our results were summarized in a mechanistic dynamic model of the gating mechanism of the receptor.

Nevertheless, many questions remain open, the deepest of which relates to the real gating mechanism of the human NMDAR. How is allosteric communication transmitted from the binding sites of ligands to the ATD and TMD? This suggests a different role of glutamate and glycine in the activation mechanism. The binding of one ligand may induce a conformation adapted to the binding of the other ligand. Which ligand binds to the receptor first, and what is the role of this binding? Additional theoretical studies of the binding of each ligand to NMDAR will be required to affirm any hypothesis.

MD simulations with longer time scale (at the level of milliseconds) will be crucial to observe the channel gating in full. This study will open the door to different applications—structure-based drug design, the modeling of cation flow or a search for NMDAR interactions using presynaptic terminals.

## Material and methods

### The target and templates sequences

The protein primary sequences of the human NMDAR (hNMDAR,) were retrieved from the UniprotDatabase (http://www.uniprot.org/uniprot). The GluN1-4a and GluN2B subunit variants were used as the target sequences. The crystallographic structures, encoded in Protein Data Base (PDB) [20] as 4PE5 (resolution of 3.96 Å) [14], 4TLL and 4TLM (resolution of 3.59 and 3.77 Å respectively) [15] were chosen as the templates for homology modeling of the target. Alignment of sequences from hNMDAR (*GluN1-4a*: *Q05586-2*, *GluN2B*: *Q13224-1*), from *Rattus norvegicus* (rat) receptor (rNMDAR, template) (*GluN1*: *P35439-5*, *GluN2B*: *Q00960*) and from *Xenopus laevis* (frog) (fNMDAR, template) (*GluN1*: *Q91977*, *GluN2B*: *A9QW73*) was performed using Clustal 2.1 (http://www.clustal.org/). Identity of the human GluN1 sequence with the rat GluN1 and with the frog GluN1 sequence are 99.21% and 91.68%, respectively. Similarly, the identity of the human GluN2B sequence with the rat GluN2B and with the frog GluN2B sequence are 98.58% and 84.65%, respectively.

### Homology modeling

The PDB crystal structures 4PE5, 4TLL and 4TLM are molecular complexes composed of NMDAR and ligands: 4TLL (fNMDAR with ligands 4-[(1R,2S)-3-(4-benzylpiperidin-1-yl)-1-hydroxy-2-methylpropyl]phenol (QEM), N-acetyl-D-glucosamine (NAG), *trans*-1-aminocyclobutane-1,3-dicarboxylic acid (JEG) and 1-aminocyclopropane carboxylic acid (1AC)); 4TLM (fNMDAR with QEM, NAG, JEG and 1AC) and 4PE5 (rNMDAR with 4-[(1R,2S)-2-(4-benzylpiperidin-1-yl)-1-hydroxypropyl]phenol (QEL), NAG, tungsten ion (W^6+^), β-D-mannose (BMA), α-D-mannose (MAN), glutamic acid (E) and glycine (G)). All ligands were deleted and the proteins were used as template structures. The 3D model of hNMDAR was constructed by homology modeling using MODELLER 9.14 [39]. All mutations and engineered inter-subunits disulfide bonds, introduced to rigidify the protein, were replaced by the native residues. A hundred of independent models were generated and optimized with the method VTFM using conjugate gradients with a maximum iteration number of 300. The degree of the VTFM was set by the *autosched* module applying the long, thorough optimization schedule of *autosched*.*slow* to avoid knotted loop formation in the long, neighboring, missing loop regions of the template structures. Subsequently the models were refined by short molecular dynamics (MD) simulations (of 2 ns) and by simulated annealing using the predefined function, *refine*.*slow* of the *refine* module of MODELLER. Optimization of each model was repeated two times with an objective function cutoff of 10^6^. Further, the loop refinement procedure employing the *loopmodel* module of MODELLER was performed. The loop refinement level was set by the predefined function, *a*.*loop*.*md_level* to *refine*.*fast*. The generated 3D models with correct loop topology (no structural aberrations, i.e., intramolecular "nodes") were validated by MolProbity 4.1. Based on all-atom contacts and protein geometry criteria, the model with the lowest MolProbity score (Ramachandran plot) was selected as a pertinent structural model of hNMDAR. Sidechain protonation states of this model were assigned by visual inspection and calculations with the PropKa 3.1 [40]. The constructed model of non-bound receptor (hNMDAR) was used for generation of the ligands-bound hNMDAR complex (hNMDAR•G•E).

### Molecular docking

The coordinates of the glutamate (E) and glycine (G) structural models were taken from the 4PE5 crystal structure. The atomic coordinates of spermine were retrieved from PDB structure 3C6K and the atomic coordinates of PYD-106 were generated from the 2D structural formula with utility Chemdoodle.com. The 3D models of E and G, treated as zwitter ions with ionized side-chains, were docked into the generated model of hNMDAR. Spermine and PYD-106 were docked into the two representative conformations, cleft-closed ATD and cleft-open ATD, of the unbound NMDAR and one representative conformation of the bound receptor. Docking of each molecule into the targets was performed using AutoDock 4.2 [41]. The docking sites of E and G were obtained by superimposition of the hNMDAR model with the crystallographic structure 4PE5. The coordinates of the Cα atom of E and G from 4PE5 were used as centers for the search of the binding sites. The number of grid points were chosen as 70, 70, 70 in X, Y, Z directions with a grid spacing of 0.375 Å. 1,000 independent docking poses were generated for each ligand using the Lamarckian genetic algorithm (LGA) method with a maximum number of 2,500,000 energy evaluations, maximum number of 27,000 generations and a population size of 150. The ligands and the side-chains of the binding residues were set to be flexible. The docking poses of spermine and PYD-106 were ranked by the binding free energies estimations (score function) and the population. The docking poses of G and E were analyzed by the clustering method from the RMSD matrix of ranking solutions and ranked by the binding free energies estimations (score function). RMSDs were calculated between the binding sites (binding residues and ligand) of the docking models and that of the highly resolved X-ray structure (PDB id: 5H8Q) presenting the separate ligand binding domain (LBD) complexed with E and G. The docking poses with lowest RMSDs and lowest binding free energies were selected as final conformations. The protonation states of the titratable residues were determined by PropKa 3.1 and assigned after visual inspection. The aspartate residues in the binding sites were considered as ionized. The distances between each ligand and its binding residues were monitored during each simulation. The contacts between each ligand and protein were conserved throughout all the simulations, with the exception of one trajectory, where after 160 ns one of the E moved from its binding position and was partially unbound (not shown). Therefore the second segment (after 160 ns) of this trajectory was not considered for data analysis.

### Systems modeling

A pre-equilibrated 1-palmitoyl-2-oleoyl-D-glycero-3-phosphatidylcholine (POPC) bilayer consisting of 1,225 lipid molecules was packed around the TMD of hNMDAR using the *Membrane Builder* facility of CHARMM-GUI [42]. Two generated models, hNMDAR and hNMDAR•G•E, were inserted into the membrane. The receptor was oriented with respect to membrane in a way that the charged side chains of the TMD were surrounded by either the lipid head groups or the water molecules. The membrane position relative to the receptor was determined based on the database of Orientations of Proteins in Membranes (OPM) [43]. Both receptor-membrane systems were solvated with 226,708 TIP3P [44] water molecules resulting in 210.125×210.125×215.824 Å^3^ simulation box cells with a distance of at least 20 Å between protein surface and box face. Each box was replicated by periodic boundary conditions. The sodium and chloride ions corresponding to a physiological ion strength of 150 mM were added. Additional sodium counterions were added to achieve a neutral net charge of the systems. The total number of atoms in hNMDAR and in hNMDAR•G•E was 897,124 and 897,188 respectively.

### Systems energy minimization/set-up of the system

The minimization procedure and set-up of molecular dynamics (MD) simulations were performed with GROMACS 5.0.4 program package [45] using the CHARMM-36 all-atom parameter set [46]. The real space summation of electrostatic interactions was truncated at 12 Å, and the Particle Mesh Ewald (PME) method was used to calculate the electrostatic interactions beyond 12 Å with a grid spacing of 1.6 Å and an interpolation order of 4. Van der Waals interactions were calculated using a cut-off of 12 Å. The solvated systems were energy minimized to eliminate unfavorable positions. Harmonic positional restraints were applied on protein, ligands, lipids and dihedral restraints on lipid heavy atoms to achieve smooth minimization. First 6,000 steps steepest descent algorithm was used. The harmonic force constants were decreased every 1000 steps, adopting the values for protein backbone/side-chain atoms 4,000/2,000, 2,000/1,000, 1,000/500, 500/200, 200/50, 50/0 kJmol^-1^nm^-2^; for the ligands atoms − 4,000, 2,000, 1,000, 500, 200, 50 kJmol^-1^nm^-2^; for the lipid phosphor (P) atom in Z direction (orthogonal to the membrane) − 1,000, 1,000, 400, 200, 40, 0 kJmol^-1^nm^-2^; for the improper dihedral angle formed by the glycerol carbon and the oleoyl ester oxygen atoms of POPC (restricted to 120°) and for the dihedral angle around the double bond of the oleoyl chain of POPC (restricted to 0°) − 1,000, 400, 200, 100, 0 kJmol^-1^rad^-2^. Subsequently unconstrained minimization was applied for 50,000 steps with steepest descent method.

The minimized systems were equilibrated over 2 ns (*NVT*) and 13 ns (*NPT*). Similarly to the energy minimization procedure, gradually decreasing harmonic restraints were applied on the protein, ligands and the lipid heavy atoms. The force constant values were decreased every 1 ns for the first 6 ns of the equilibration according to the following procedure: the protein backbone/side-chain atoms − 4,000/2,000, 2,000/1,000, 1,000/500, 500/200, 200/50, 50/0 kJmol^-1^nm^-2^; the ligands atoms − 4,000, 2,000, 1,000, 500, 200, 50 kJmol^-1^nm^-2^; the lipid phosphor (P) atom in Z direction − 1,000, 1,000, 400, 200, 40, 0 kJmol^-1^nm^-2^; improper dihedral angle formed by the glycerol carbon and the oleoyl ester oxygen atoms of POPC (restricted to 120°) and dihedral angle around the double bond of the oleoyl chain of POPC (restricted to 0°) − 1,000, 400, 200, 100, 0 kJmol^-1^rad^-2^.

### Production of MD trajectories

All-atom MD simulations were performed on 512 nodes of HP supercomputer CURIE at GENCI with CHARMM-36 force field integrated in GROMACS 5.0.4 package. Three independent of 300 ns production trajectories were performed for each system, using 3 different equilibrated conformations. For each trajectory new initial random velocities were generated. The following MD protocols were used: the integration time step was 2 fs; the isobaric–isothermal (*NPT*) ensemble was employed; the pressure was set to 1 bar using semi-isotropic coupling (uniform scaling of X-Y box vectors, independent Z) to the Parrinello-Rahman barostat with a time constant of 2 ps and an isothermal compressibility of 4.5*10^−5^ bar^−1^; the temperature was kept constant at 310 K using the Nosé-Hoover thermostat with a time constant of 0.5 ps. All bonds were constrained using the Linear Constraint Solver (LINCS). Atomic coordinates were recorded every 10 ps.

### Data simulation analysis

The MD trajectories were analysed (RMSDs, RMSFs, DSSPs, PCA, clustering and geometric measurements) with tools included in the GROMACS 5.0.4 package. Correlation matrices were calculated with the Bio3d R package [47]. The RMSDs were calculated on 3 × 30,000 conformations generated over the 3 × 300 ns MD runs per system. Only the Cα atoms of hNMDAR were considered. According to the RMSD curves, the initial interval of each trajectory − 15, 50, 85 ns for hNMDAR and 15, 20, 25 ns for hNMDAR•G•E − were omitted as non-equilibrated. The truncated trajectories were merged for each system and used for the RMSF calculation respective the average structure, correlation analysis, PCA and clustering. To remove the overall translation and rotation, each frame of the merged trajectory was superimposed onto the Cα atoms of the *reference structure* that is the first conformation of the equilibrated merged trajectory. The Gromos algorithm of Gromacs 5.0.4 was used for clustering analysis using a cut-off of 4.5 A for the both states of hNMDAR. The middle structure of each cluster was considered as *representative conformation*. Visual inspection of molecular conformations was made with PyMOL and VMD. Graphs were generated using Grace, correlation matrix has been drawn with the Bio3d R package. To visualize the PCA eigenvectors, two extreme projections of each eigenvector along the trajectory on the averaged structure were calculated using Gromacs 5.0.4. A python script of PyMOL (Sean M. Law) was used to draw small arrows from the starting structure to the final structure. A python script (Pablo Guardado Calvo) of PyMOL was applied to calculate and represent rotation axes (big arrows) of domains between two extreme structures by aligning the structures and extracting the transformation matrix (T). Direction of the rotation axis and a point were obtained from T and used to create a cgo object representing the axis. The two extreme projections of a trajectory along a PCA vector on the average structure and interpolation of 100 frames between them was performed using *gmx anaeig* utility from Gromacs 5.0.4. Distances between facing bottleneck residues (diameters) of the TMD channel were monitored for these 100 frames to evaluate channel pore characteristics along a given PCA mode.

### Geometry measurements

To characterize the geometry of LBD throughout the simulation, geometric centers, i.e. centroids of each D1, D2 lobes and linking hinges (C1, C2 and C3, respectively) were determined. Distance C1⋯C2 and angle formed by C1, C3 and C2 were monitored during simulation. Similarly, to characterize the geometry of TMD, distances of bottleneck residues (A645 and A645’, T646 and T646’) and angles formed by each two M3 helices were monitored during the simulation. The channel profile and size of bottleneck diameter was determined in the *representative structure* of the most populated conformational cluster for both receptors using CAVER 3.0 with a probe radius of 0.5 Å, shell radius of 3.2 Å, shell depth of 4 Å and a clustering threshold of 3.5 Å.

### Distribution functions

For a given ensemble, the observed cumulative distribution was constructed by calculating the C*α*-RMSD for all conformational pairs and integrating the results over the conformers in the generated set as defined in [48].

### Surface pockets prediction

Surface pockets were identified for the *representative structures* of the two most populated clusters of hNMDAR, and the most populated cluster of hNMDAR•G•E, respectively, using *fpocket 1*.*0* [32]. A minimum radius of 5 Å, and a maximum radius of 7 Å of carbone-α atom sphere was used to filter out the pocket sizes.

## Supporting information

S1 Fig Analysis of MD simulation data. (**A**) Pearson cross-correlation map of Cα atoms displacements of hNMDAR calculated separately for only the 3rd trajectory (the lightest grey on the RMSD profile in Fig 3 of the main text.) after removing the overall rigid body motions. Correlated and anti-correlated motions between atom pairs are color-coded from red (positive) to blue (negative). (**B-C**) The 2nd and 3rd modes (PCA) of the unbound (B) and bound (**C**) receptor calculated on the Cα-atoms from the merged trajectories. Slow motions are illustrated by small arrows projected on the hNMDAR (left) and hNMDAR•G•E (right).
(TIF)

S2 Fig Contacts between the lobes R1 and R2 from ATD. Distances between centroids defined on each lobe R were monitored over the MD simulations. List of pairs of residues showed short contacts are exampled for the one heterodimer.
(TIF)

S3 Fig The LBD geometry. (**A)** Two hinged lobes, D1 and D2, which form a narrow agonist-selective binding cleft in each chain. (**B**) The LBD heterodimer interface is braced by specific contacts between the polar residues from the D1 and D2 lobes, Q696 from GluN1, N693 and N697 from GluN2B of the adjacent chains and Y535 of GluN1, with residues located at the D1-D2 hinge. (**C** and **D**) The distance between the lower and upper LBD-lobe interface residues were monitored throughout MD simulations of unbound and bound forms of hNMDA receptor. The residues numbers are shown as in the list (black) and as in the sequence (green).
(TIF)

S4 Fig Dynamical features of the TMD. The bottleneck diameters, defined as distances between the Cα atoms of A645 from two GluN2B chains (black), of T646 from two GluN1 chains (red), are shown for hNMDAR (solid lines) and hNMDAR•G•E (dashed lines). Distances were measured along the 1^st,^ 2^nd^ and 3^id^ PCA modes (for 100 frames in-between extreme projections,). Slow motions (modes 1, 2 and 3) are illustrated by small arrows projected on the hNMDAR (left) and hNMDAR•G•E (right). Two large arrows indicate rotational axes of structural blocks within a domain (rigid body motion) in LBD (orange) and TMD (blue).
(TIF)

S5 Fig (**A**) Residues R684 (GluN1) and E658 (GluN2B) localized in the interface region connecting LBD and TMD. (**B-C**) Metrics (distance and angle) describing of the shape formed by these residues along the first PCA mode (for 100 frames in-between extreme projections, see Methods).
(TIF)

S1 File Atomic coordinates of unbound hNMDAR (cluster 1) are reported in file hNMDAR_Cluster1. (PDB)

S2 File Atomic coordinates of unbound hNMDAR (cluster 2) are reported in file hNMDAR_Cluster2. (PDB)

S3 File Atomic coordinates of bound hNMDAR are reported in file hNMDAR_GE_Cluster1. (PDB)
