# Supplementary figures and images for: How does binding of agonist ligands control intrinsic molecular dynamics in human NMDA receptors?
Source: PLoS One. 2018 Aug 3;13(8):e0201234. doi: 10.1371/journal.pone.0201234 (PMC6075769; doi:10.1371/journal.pone.0201234)

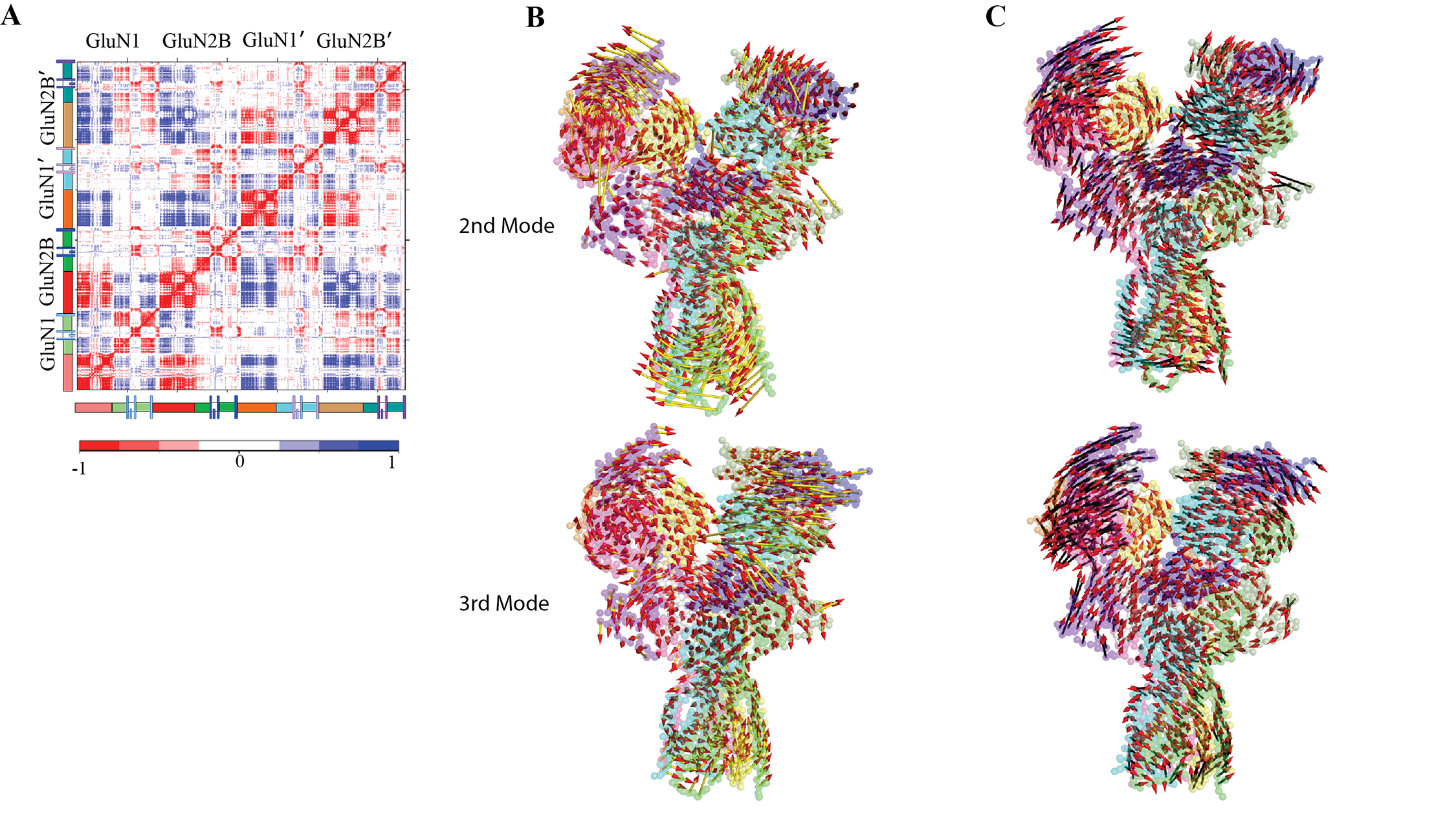

Supplement: S1 Fig — (A) Pearson cross-correlation map of Cα atoms displacements of hNMDAR calculated separately for only the 3rd trajectory (the lightest grey on the RMSD profile in Fig 3 of the main text.) after removing the overall rigid body motions. Correlated and anti-correlated motions between atom pairs are color-coded from red (positive) to blue (negative). (B-C) The 2nd and 3rd modes (PCA) of the unbound (B) and bound (C) receptor calculated on the Cα-atoms from the merged trajectories. Slow motions are illustrated by small arrows projected on the hNMDAR (left) and hNMDAR•G•E (right). (TIF) [file pone.0201234.s001.tif]

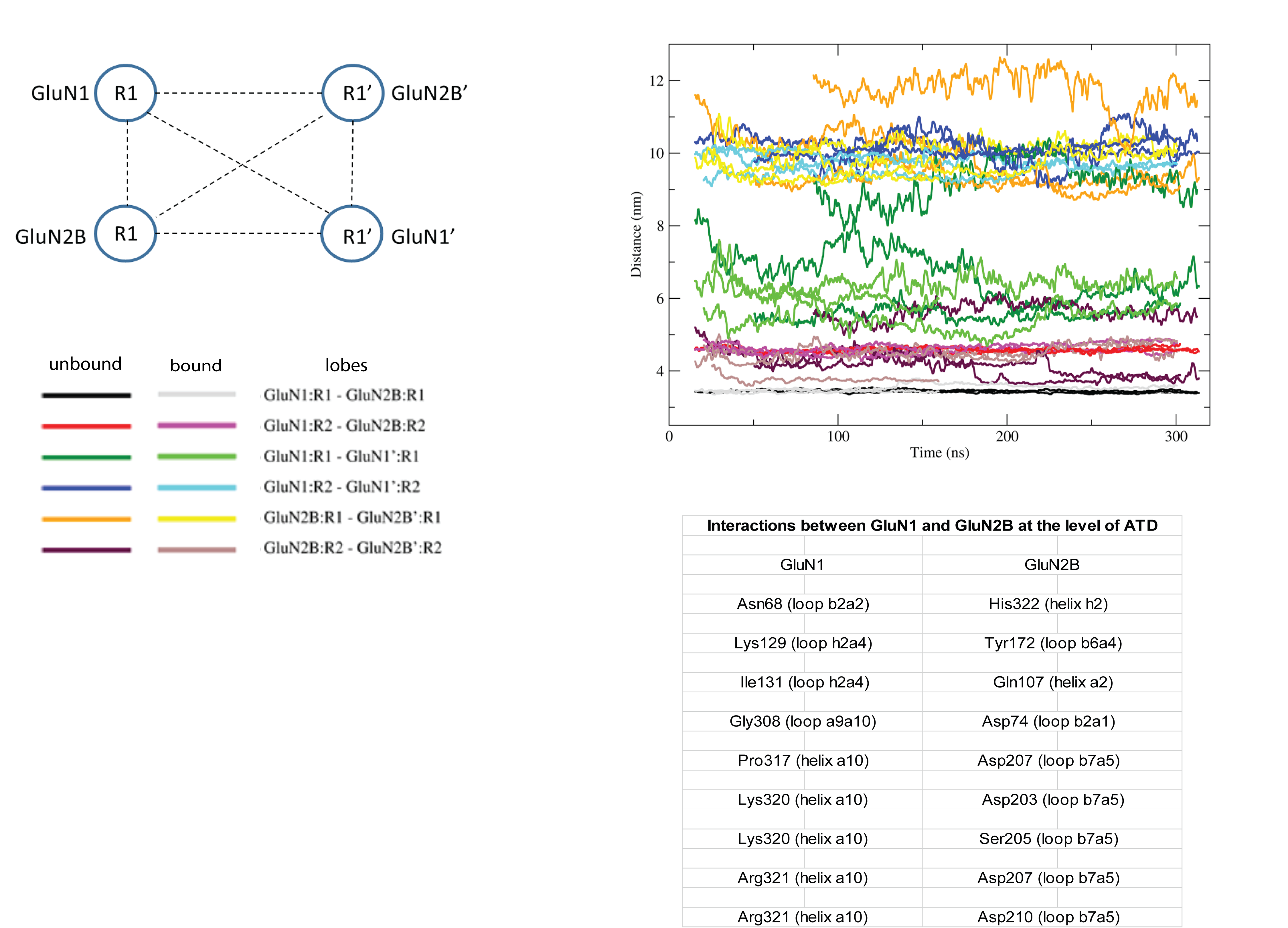

Supplement: S2 Fig — Distances between centroids defined on each lobe R were monitored over the MD simulations. List of pairs of residues showed short contacts are exampled for the one heterodimer. (TIF) [file pone.0201234.s002.tif]

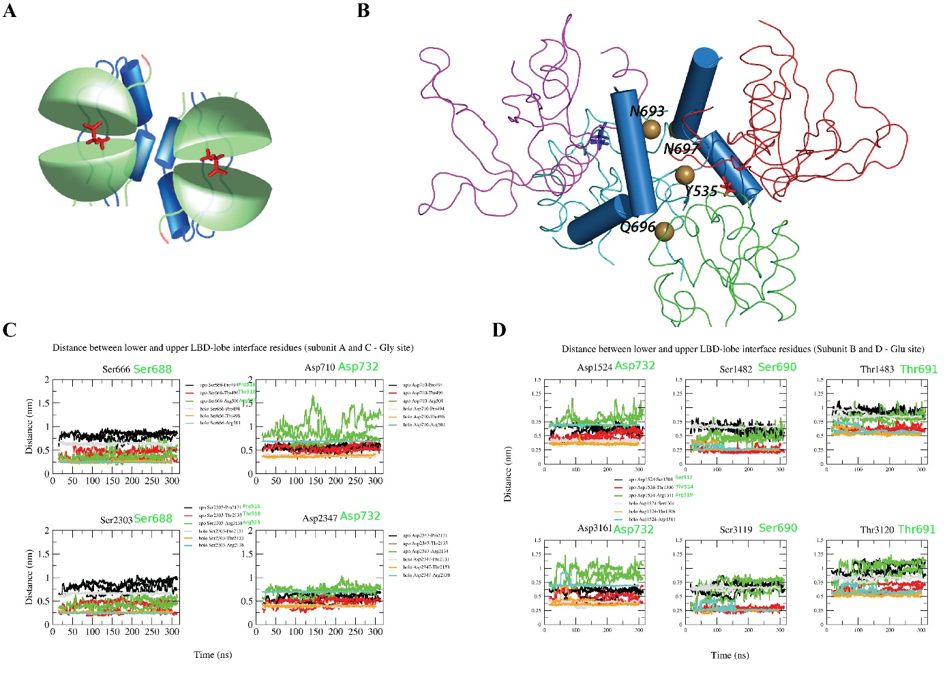

Supplement: S3 Fig — (A) Two hinged lobes, D1 and D2, which form a narrow agonist-selective binding cleft in each chain. (B) The LBD heterodimer interface is braced by specific contacts between the polar residues from the D1 and D2 lobes, Q696 from GluN1, N693 and N697 from GluN2B of the adjacent chains and Y535 of GluN1, with residues located at the D1-D2 hinge. (C and D) The distance between the lower and upper LBD-lobe interface residues were monitored throughout MD simulations of unbound and bound forms of hNMDA receptor. The residues numbers are shown as in the list (black) and as in the sequence (green). (TIF) [file pone.0201234.s003.tif]

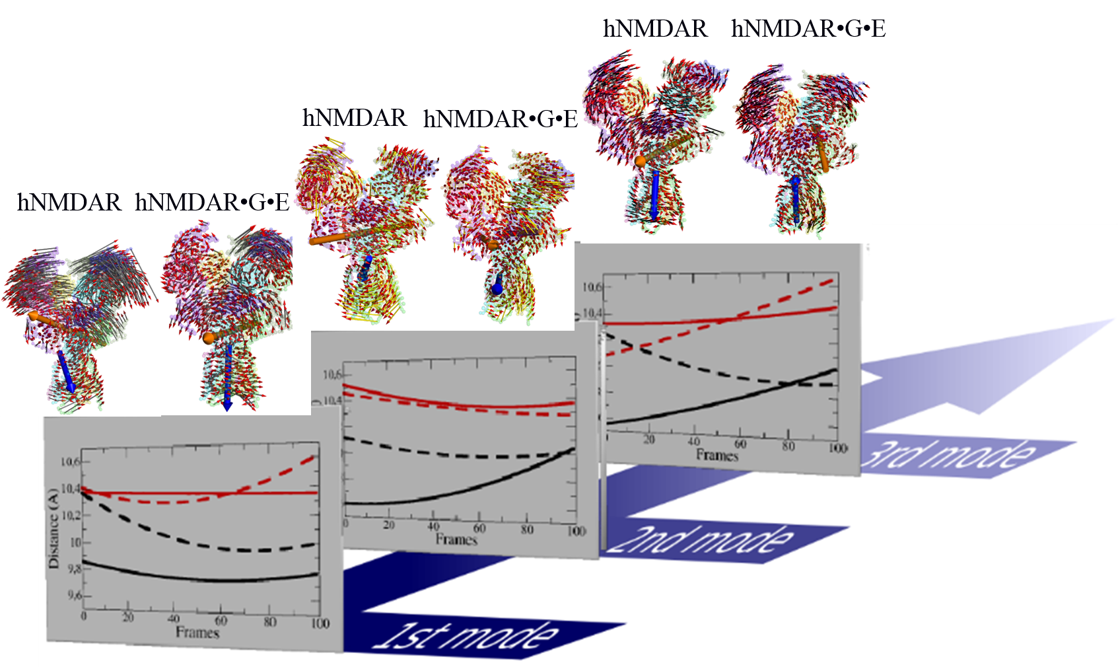

Supplement: S4 Fig — The bottleneck diameters, defined as distances between the Cα atoms of A645 from two GluN2B chains (black), of T646 from two GluN1 chains (red), are shown for hNMDAR (solid lines) and hNMDAR•G•E (dashed lines). Distances were measured along the 1st, 2nd and 3id PCA modes (for 100 frames in-between extreme projections,). Slow motions (modes 1, 2 and 3) are illustrated by small arrows projected on the hNMDAR (left) and hNMDAR•G•E (right). Two large arrows indicate rotational axes of structural blocks within a domain (rigid body motion) in LBD (orange) and TMD (blue). (TIF) [file pone.0201234.s004.tif]

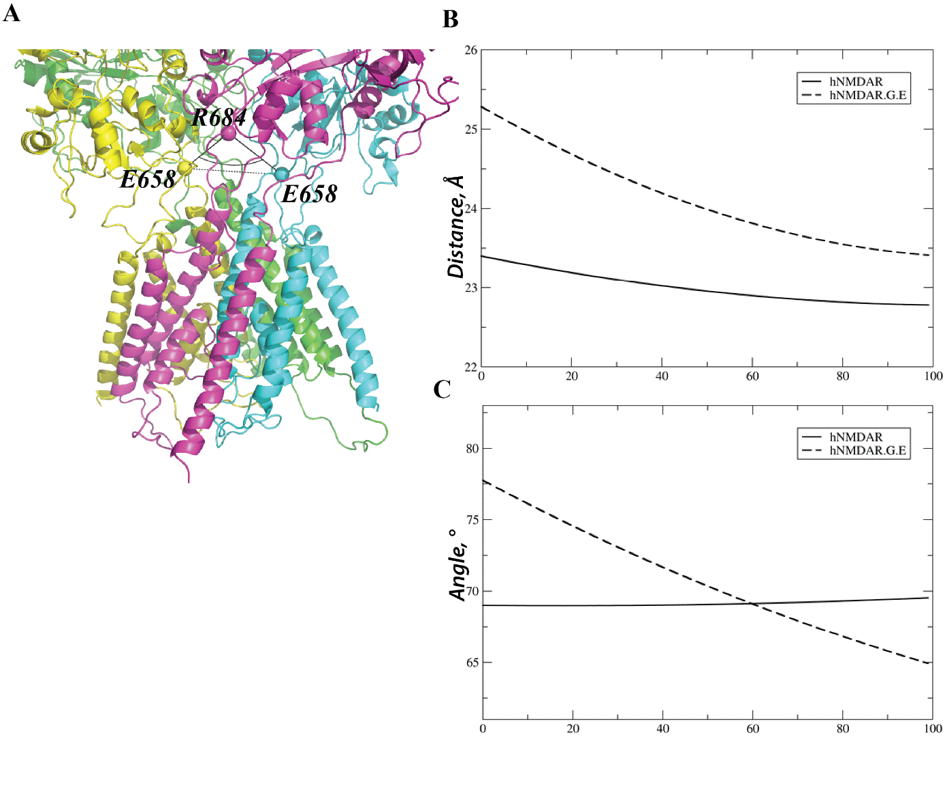

Supplement: S5 Fig — (A) Residues R684 (GluN1) and E658 (GluN2B) localized in the interface region connecting LBD and TMD. (B-C) Metrics (distance and angle) describing of the shape formed by these residues along the first PCA mode (for 100 frames in-between extreme projections, see Methods). (TIF) [file pone.0201234.s005.tif]
